# A high-throughput chemical screen with FDA approved drugs reveals that the antihypertensive drug Spironolactone impairs cancer cell survival by inhibiting homology directed repair

**DOI:** 10.1093/nar/gku217

**Published:** 2014-03-25

**Authors:** Or David Shahar, Alkmini Kalousi, Lital Eini, Benoit Fisher, Amelie Weiss, Jonatan Darr, Olga Mazina, Shay Bramson, Martin Kupiec, Amir Eden, Eran Meshorer, Alexander V. Mazin, Laurent Brino, Michal Goldberg, Evi Soutoglou

**Affiliations:** 1 Department of Genetics, Alexander Silberman Institute of Life Sciences, Hebrew University of Jerusalem, Jerusalem 91904, Israel; 2 Institut de Génétique et de Biologie Moléculaire et Cellulaire (IGBMC), UMR 7104 CNRS, UdS, INSERM U964, BP 10142, F-67404 Illkirch Cedex, CU de Strasbourg, France; 3 Department of Biochemistry and Molecular Biology, Drexel University College of Medicine, Philadelphia, PA 19102, USA; 4 Department of Molecular Microbiology and Biotechnology, Tel Aviv University, Ramat Aviv 69978, Israel

## Abstract

DNA double-strand breaks (DSBs) are the most severe type of DNA damage. DSBs are repaired by non-homologous end-joining or homology directed repair (HDR). Identifying novel small molecules that affect HDR is of great importance both for research use and therapy. Molecules that elevate HDR may improve gene targeting whereas inhibiting molecules can be used for chemotherapy, since some of the cancers are more sensitive to repair impairment. Here, we performed a high-throughput chemical screen for FDA approved drugs, which affect HDR in cancer cells. We found that HDR frequencies are increased by retinoic acid and Idoxuridine and reduced by the antihypertensive drug Spironolactone. We further revealed that Spironolactone impairs Rad51 foci formation, sensitizes cancer cells to DNA damaging agents, to Poly (ADP-ribose) polymerase (PARP) inhibitors and cross-linking agents and inhibits tumor growth in xenografts, in mice. This study suggests Spironolactone as a new candidate for chemotherapy.

## INTRODUCTION

The most severe type of DNA damage is DNA double-strand breaks (DSBs). Although our cells are constantly challenged by DNA damage that is commonly repaired by non-homologous end-joining (NHEJ), if not properly repaired, they can lead in some cases to complex chromosomal translocations, cell death and cancer (1,2). Though the last may be a rare event, cells harboring mutations, which lead to uncontrolled cell cycle, have a selective advantage, and therefore might lead to tumors. Two major repair mechanisms have evolved to repair DSBs: NHEJ and homologous recombination (HR) based repair (3,4). NHEJ can lead to an inaccurate repair as it may involve processing of the DNA ends at the site of breakage. Homology directed repair (HDR) is considered accurate, since it relies on the sister chromatid as a template, and can therefore, mainly be employed during or after S-phase of the cell cycle. During HDR, DNA is processed to generate single-stranded ends that are coated by Replication Protein A (RPA) and subsequently by RAD51 filaments. These nucleoprotein filaments are then prone to invade the homologous strand so that subsequent repair by HDR can take place (5,6,7).

In mammals, HDR occurs at a much lower frequency than NHEJ (3,4,8,9,10,11,12,13,14,15,16). The rare occurrence of HDR may result in a less accurate DSB repair and is probably the reason for low efficiency of gene targeting used in research and disease therapy. Deciphering the molecular mechanism of HDR, including the components it comprises and their modes of action, is key to understanding how it can be potentially modulated for purposes of disease management and research.

High-throughput screening approaches have been mostly confined to searching for genes, which modulate HDR efficiency (17,18,19,20). Such screens are generally based on siRNA techniques and often result in a relatively high false positive rate due to non-specific silencing of genes, as was recently demonstrated (18). Extending the search to identifying chemical compounds that modulate HDR would be more applicable in a clinical setting, for example, to decrease HDR prevalence in cancer chemotherapy or increase HDR frequency to improve gene targeting efficiency. Previous high-throughput screens for revealing chemical components that modulate HDR have been limited to *in-vitro* assays, by assessing Rad51 activity, Rad51 ability to bind single-stranded DNA or RAD54 branch migration (21,22,23,24,25).

Here, we developed a robust high-throughput screening methodology with only negligible variance levels. We introduced DSBs in populations of cells with similar kinetics and efficiency and screened for chemical compounds that modulate DSB repair by HDR. We identified and validated three compounds that significantly affect HDR efficiency, two of them Idoxuridine and retinoic acid (RA) augmented HDR, while Spironolactone (Spiro) repressed HDR. We focused on Spiro and found that in addition to inhibition of HDR, it sensitizes cells to radiomimetic drugs and PARP inhibitors and cross-linking agents and it inhibits tumor growth in xenografts in mice. We found that Spiro reduces RAD51 foci formation but does not act on resection. It should therefore be considered as potential chemotherapy agent, especially for patients who do not respond well to existing therapy.

## MATERIALS AND METHODS

### Cell culture

Cells were cultured in Dulbecco's modified Eagle's medium (DMEM) supplemented with 10% fetal calf serum (FCS), except for U2OS-DRGFP and HRind cells that were cultured in Phenol red-free DMEM that lacks phenol red and with charcoal treated FCS, L-glutamine 20 mM, penicillin 500 units/ml and streptomycin 0.5 mg/ml (Biological Industries, Beit Haemek, Israel) at 37ºC and 5% CO2. U2OS-DRGFP and HRind cells were maintained with 1 μg/ml puromycin or 0.2 mg/ml G418 and 1 mg/ml puromycin, respectively.

### Screen

Screening was performed at the High Throughput Screening facility of the Institut de Génétique Et de Biologie Moléculaire et Cellulaire, using the Prestwick Chemical Library® (http://www.prestwickchemical.fr/) containing 1200 small molecules that are approved drugs. The screen was performed in 96-well cell culture microplates with a particular focus on avoiding microplate edge effects. On day 1, 7500 HRind cells were seeded per 0.34 cm^2^ (96-well) in 180 μl cell culture medium (Phenol red-free DMEM supplemented with 4.5 g/l glucose, charcoal-treated FCS 10%, 400 G418 Puromycine) in the presence of small molecule compounds (20 μM final concentration, Dimethyl sulfoxide (DMSO) concentration of 0.2% v/v). Cells were then incubated at 37°C, 5% CO2. Four hours after cell seeding, Triamcinolone Acetonide (TA, 10^−7^ M final concentration) was added to promote mCherry-I*SceI*-GRLBD nuclear translocation. Controls were DMSO alone (0.2% v/v), DNA-PKi, NU7026 (20 μM in DMSO 0.2% v/v) or ATMi, Ku55933 (10 μM in DMSO 0.2% v/v). On day 4 (two and a half day after TA addition), cells were washed, ﬁxed with 1.5% paraformaldehyde, permeabilized with 0.5% Triton X-100, stained with 4',6-diamidino-2-phenylindole (DAPI) (1 μg/ml). The screens were performed in a TECAN robotic station (for cell handling, treatment and staining) and to a Caliper Twister II robotic arm coupling microplate stacks to the INCell1000 analyzer microscope (GE LifeSciences).

### Statistical analysis of the screen

The Multi Target Analyzer software (GE LifeScience) was used for a four steps analysis based on the shape (form factor), size and intensity of nuclei fluorescent stain DAPI, and on the intensity of nuclear mCherry staining to select positive U2OS reporter cells having a well response to TA treatment with nuclear translocation of the I*SceI* endonuclease fused to the glucocorticoid receptor ligand-binding domain (GRLBD) and mCherry protein (mCherry-I*SceI*-GRLBD). DAPI was first used to find nuclei corresponding to individual units considered by the analysis software. We then selected mCherry positive cells using a threshold of Nuc/Cyto intensity for mCherry intensity signal.

Then, we analyzed the DAPI digital signal to exclude dead cells and their potential non-specific signal in both Green Fluorescent Protein (GFP) and mCherry channels. This exclusion was performed first using the nuclei form factor parameter (called « Nuc1/form factor » by keeping cells for which this parameter is below 1.48. The « Nuc1/form factor » parameter corresponds to the mean nucleus roundness index, its value ranging from 1 to infinity, where 1 is a perfect circle (« Nuc1/form factor » = perimeter^2^/(4*p*Area)). Then we excluded cells having the Nuclear Intensity Coefficient of Variation (CV) parameter above 0.19. This parameter corresponds to the coefficient of variation of the intensity of pixels over the population of pixels comprising the nuclear region, meaning that we excluded cells undergoing the characteristic apoptotic nuclei condensation. Finally, the GFP nuclear signal was analyzed to determine the efficiency of HR as reported by the DR-GFP I*sceI* construct. Following image analysis, the RReportGenerator software was used to determine control-based normalized HDR frequencies in treated and selected cells (26). A hit was defined as activator or repressor when its normalized value deviated more (for up-regulators) or less (for down-regulators) than 4 SD from the control DMSO- and TA-treated cells mean value.

### HDR flow cytometry based assays

Activation of the HRind cells was done by addition of 10^−7^ M Dex (Sigma, Aldrich) and of the U2OS-DRGFP cells by I*SceI* infection, using adenovirus (provided by F. Graham), similarly to (18). Analyzing GFP-positive cells out of the mCherry-positive cells in ﬂow cytometric analysis was performed using FACSAria Cell-Sorting System (Becton Dickinson), similarly to Sartori *et al.* (33). For each compound, the background was set in the absence of DSB induction and reduced using the same gate. Sample analysis was done in FlowJo program.

### Cell-cycle analysis

Cells were incubated with the different compounds or DMSO for 24 h and stained with 5 mg/ml Hoechst-33342 (Molecular Probes, Eugene, OR, USA) for 20 min. Following extraction, cells were analyzed using ﬂow cytometry (SORP LSRII analyzer) for DNA content (UV 355 nm, 60 mW laser) and mCherry (561 nm, 25 mW laser) using Cellquest or FlowJo programs. Doublets were discriminated as described (27). Gating was set for DMSO and the same gate was used for all the treatments. For statistical analysis a paired, two tailed T test was performed using the Prism software (Graphpad, CA, USA).

### Cell survival assays

For survival assays, U2OS cells were plated in 6-well plates in triplicates (500 cells per well) and were subsequently treated with Spiro (10 μM) or DMSO for 24 h. The next day the cells were treated with increasing concentrations of either Phleomycin or the PARP inhibitors ABT888 and KU0058684 or Mitomycin C (MMC) or Cisplatin. The cells were treated with Phleomycin and Cisplatin for 1 h, with MMC for 16 h, and then they were released with the addition of fresh medium containing Spiro or DMSO. Conversely, in the case of PARP inhibitors the cells were treated throughout the whole survival assay in medium containing the appropriate PARP inhibitor combined with Spiro or DMSO. Subsequently, the cells were let to grow in colonies for 10 days. After 10 days, the cells were washed with phosphate buffered saline, fixed with 4% Formaldehyde for 30 min and stained with Crystal Violet (0,1% w/v) for 1 h. The number of colonies per well was measured using the ImageJ software.

### *In vivo* tumor growth assay

Experiments were approved by the Israeli Animal Care and Use Committee (NS-13–13812–4). Mice were kept in Specific Pathogen free (SPF) approved facility. For xenografting, weaned Nod-SCID males aged 8 weeks were sedated with Isoflurane. Using a 27G needle 10^6^ cells suspended in a total volume of 100 μl growing media were injected subcutaneously to each flank. Following tumor establishment, mice were divided into two groups (three mice in each) and treated at a double-blind procedure, with 25 μg/g body weight of Spiro or DMSO, three times per week. Tumor volume was measured concurrently using a caliper and tumor volume was calculated using the modified ellipsoidal formula. Mice were weighed to monitor any changes in body mass and their general health examined throughout the duration of the experiment.

### Antibodies for immunofluorescence and western blots

For immunofluorescence, U2OS cells were grown on cover-slips, the cells were incubated with DMSO or Spiro (40 μM) for 24 h and then mock or Phleomycin (10 μg/ml)-treated for 1 h. For recovery the cells were grown for 2, 6 or 24 h in the presence of DMSO or Spiro (40 μM). In the case of U2OS lacO/I*SceI*/tet19 cells (U2OS 19), the cells were co-transfected with the plasmid vectors mCherryLacI and HA-I*SceI* or empty vector and were grown in medium containing Spiro or DMSO for 24 h. Subsequently, the cells were fixed with 4% formaldehyde and permeabilized with 0.5% Triton X 100. Antibodies used are rabbit anti-53BP1 (Bethyl Laboratories), mouse anti-phospho-Histone H2A.X (Ser139), clone JBW301 (Merck Millipore), rabbit anti-RAD51 PC130 (Calbiochem), rabbit anti-BRCA1 sc642 (Santa Cruz), mouse anti-CtIP clone 14–1 (Active Motif) and mouse anti-RPA32 NB600–565 (Novus). For western blots, U2OS cells were lysed in RIPA buffer, the protein content was quantified by Bradford and the samples were analyzed by sodium dodecyl sulphate-polyacrylamide gel electrophoresis. Antibodies used are rabbit anti-phospho-Histone H2A.X (Ser139) ab2893 (Abcam), rabbit anti-H2A.X ab11175 (abcam), rabbit anti-phospho-RPA32 S4/8 A300–245 (Bethyl), mouse anti-RPA32 NB600–565 (Novus) and mouse anti-alpha-tubulin T9026 (Sigma).

### D-loop assay

The effect of Spiro on the RAD51 strand exchange activity was measured using the D-loop assay (28,29). Human RAD51 (0.3 μM) was incubated with Spiro (at indicated concentrations) in buffer containing 25 mM Tris-acetate (pH 7.5), 1 mM ATP, 1 mM CaCl_2_, 100 μg/ml BSA, 2 mM DTT, and 20 mM KCl (added with the protein stock) and 2% v/v DMSO (added with Spiro) for 10 min at 37°C. Then ^32^P-labeled 90-mer ssDNA (oligo#90) (0.9 μM, nt) was added and the nucleoprotein filaments were formed for 15 min at 37°C. D-loop formation was initiated by addition of pUC19 supercoiled dsDNA (15 μM, nt) and carried out for 15 min at 37°C. The DNA products were deproteinized by treatment with 1 mg/ml proteinase K in stop mixture containing 1% SDS, 6% glycerol and 0.01% bromophenol blue for 15 min at 37°C, and analyzed by electrophoresis in a 1% agarose gel in 1XTAE buffer (40 mM Tris acetate, pH 8.3 and 1 mM EDTA) at 5 V/cm for 1.5 h. The gels were dried on DEAE-81 paper (Whatman) and quantified using a Storm 840 PhosphorImager and ImageQuant 5.2 (GE Healthcare) (28,29).

## RESULTS

**Figure 1.**
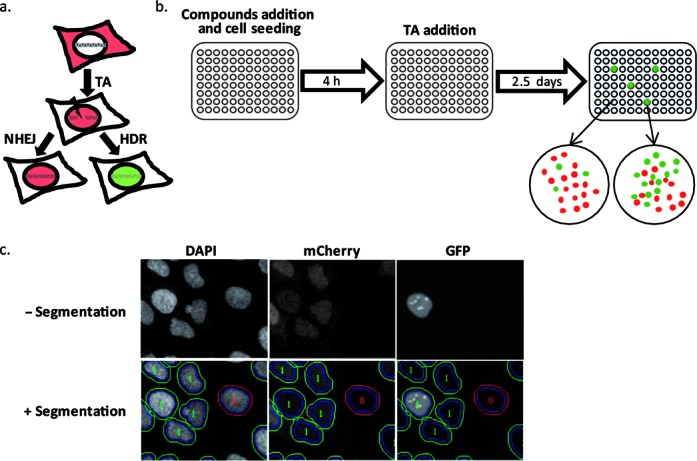
Design of the high-throughput screen for compounds that modulate HR frequencies. (**a**) A schematic representation of the HRind system. The HRind cells are U2OS cells with an integrated DRGFP cassette that constantly express mCherry-I*SceI*-GRLBD, which is localized exclusively in the cytoplasm. Upon the addition of the ligand TA, mCherry-I*SceI*-GRLBD enters the nucleus, and the endonuclease induces a DSB at the DRGFP cassette. Repair by HDR can result in GFP expression. (**b**) Illustration of the high-throughput screen for chemicals that modulate HDR. Cells were plated in 96-well plates and incubated with different compounds, from the Prestwick library. Four hours after cell seeding, TA was added for the induction of a DSB at the DRGFP cassette. The cells were fixed, stained with DAPI and subjected to an automated fluorescence microscopy after 2.5 days. In each well, a fraction of the cells expresses GFP (green, the DSB was repaired by HDR) while most cells are red (no HDR repair). (**c**) High-throughput picture analysis of the screen. The first step consisted of detecting the cell nucleus using the DAPI staining and by applying size and form limitations. Then the Analyzer software (GE Healthcare) was used to draw a ring around the nucleus corresponding to a part of the cytoplasm. By comparing the mCherry intensity between the cytoplasmic ring and the nuclear ring, the translocation of mCherry-I*SceI*-GRLBD from the cytoplasm to the nucleus was estimated for each cell. Then, cells for which there was no mCherry translocation (segmented in red) were rejected. Cells, which responded to the TA treatment (segmented in green), were selected for further analysis. For the selected cells, the nuclear GFP signal was measured.

To identify chemical compounds that alter HDR in human cancer cells, we performed a high-throughput screen of the Prestwick library, which includes 1280 approved drugs (FDA, EMEA and other agencies), using the direct repeat green fluorescent protein (DR)-GFP cassette system that enables detection of HDR (30). This recombination assay relies on the DRGFP sequence, which contains two mutated GFP genes oriented as direct repeats and separated by a drug selection marker (30). The upstream (5′) GFP gene (cassette I) carries a recognition site for the meganuclease I*SceI*, a rare-cutting endonuclease that does not cleave several eukaryotic genomes tested. The downstream (3′) GFP (cassette II) is inactivated by upstream and downstream truncations, leaving only ∼502 bp of GFP (30). Expression of I*SceI* leads to formation of DSBs and GFP reconstitution is a marker of HDR efficiency. We used the HR inducible (HRind) cells that we previously established (8), in which I*SceI*-induced DSB within the DRGFP cassette is rapidly inflicted upon the addition of an external ligand. In this system, I*sceI* is stably expressed as a chimera protein fused to mCherry and to Glucorocticoid Receptor ligand-binding domain (mCherry-I*SceI*-GRLBD). The ligand induces the translocation of the already expressed, fluorescently labeled, I*SceI* from the cytoplasm into the nucleus. This multi-color ‘switch on’ inducible cell-system obviates the need for transfections, greatly reducing cell-to-cell variability (Figure 1a) (8).

The HRind cells were plated in 96-well plates and incubated with the different compounds in triplicates. A DSB was induced at the DRGFP cassette using TA that induces nuclear entry of mCherry-I*SceI*-GRLBD (Figure 1a and (8)). Following 2.5 days of incubation, the cells were fixed, stained with DAPI and subjected to an automated fluorescence microscopy to determine the fraction of GFP-positive cells (Figure 1b and c). False positive hits due to non-specific signals or auto-fluorescence are a major caveat in high-throughput screens. To overcome this, we developed a protocol that allows focusing only on the cell population of interest and therefore minimizes false positives (see Materials and Methods). DAPI staining was used to determine nuclei segmentation and shift outliers according to the size and form of the nuclei. To determine the cells in which the endonuclease entered the nucleus and thus induced a DSB, the ratio of nuclear to cytoplasmic mCherry (from mCherry-I*SceI*-GRLBD) signal was measured, and only cells in which the ratio was higher than 1.05 were considered. The nuclear GFP intensity in these cells was measured and the ratio between the GFP-positive to GFP-negative cells was determined (Figure 1b and c).

**Figure 2.**
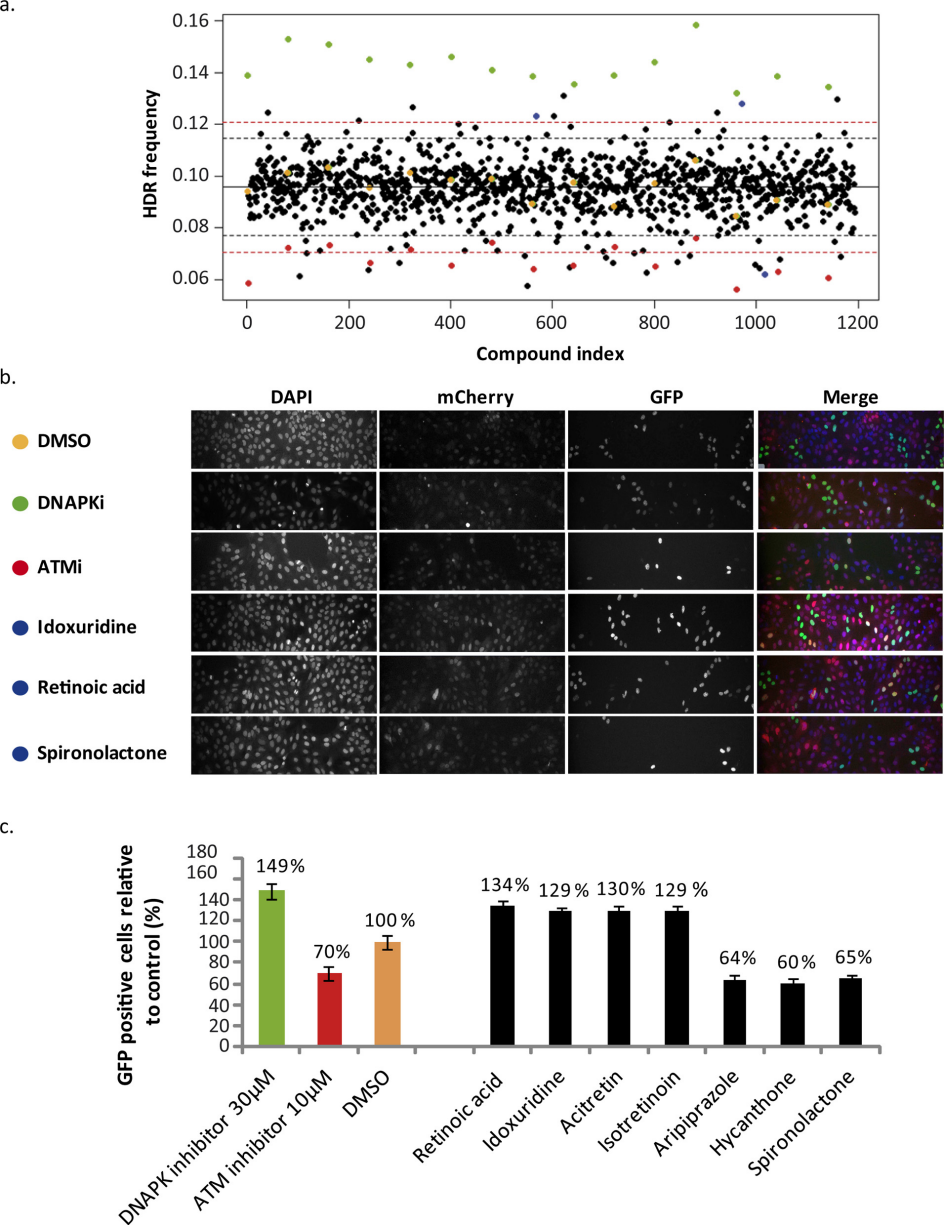
Compounds that modulate HDR frequencies. (**a**) HDR frequencies obtained from the 1280 compounds and controls (DMSO), a compound that augments HDR (Nu7026) and a compound that reduces HDR (Ku55933). Each dot represents the average of three replicas (three different plates), which each included 12 fields. The compounds are shown in alphabetical order, while repeats of the controls, which were found in each plate (DMSO in orange, Nu7026 in green and Ku55933 in red), are scattered. The broken black and red lines indicate 3 SD and 4 SD, respectively, from the mean of HDR frequency achieved with controls (DMSO). Indicated in blue are the three validated compounds (Idoxuridine, RA and Spiro). (**b**) Representative images taken by the automatic screening platform. The different compounds, in which the cells were incubated, are indicated. (**c**) Quantification of the HDR events for potential candidates. HDR frequencies of controls and the compounds which obtained 4 SD in the screen and were verified by observing their images are shown.

Each plate contained mock (DMSO), as well as control compounds that are known to augment or reduce HDR, Nu7026 and Ku55933, respectively. As expected, DMSO did not affect HDR, whereas Nu7026 and Ku55933 elevated and decreased HDR, respectively, with very small variation between replicates (Figure 2). The threshold for potential hits was 4 SD, since HDR frequencies are very low in human cells (Figure 2a). The primary screen revealed four compounds (RA, Idoxuridine, Acitretin and Isotretinoin) that increased HDR and three compounds (Spiro, Aripiprazole and Hycanthone) that decreased HDR (Table S1). Interestingly, out of the four compounds found to elevate HDR, three have a similar chemical structure and belong to the retinoid family (RA, Acitretin and Isotretinoin; Figure 2c, Supplementary Figure S1a) and, both Spiro, which was found to decrease HDR, and Megesterol Acetate (MegAc*)*, which decreased HDR by more than 3 SD, are steroids with a similar structure (Figure 2c, Supplementary Table S1 and Figure S1b).

**Figure 3.**
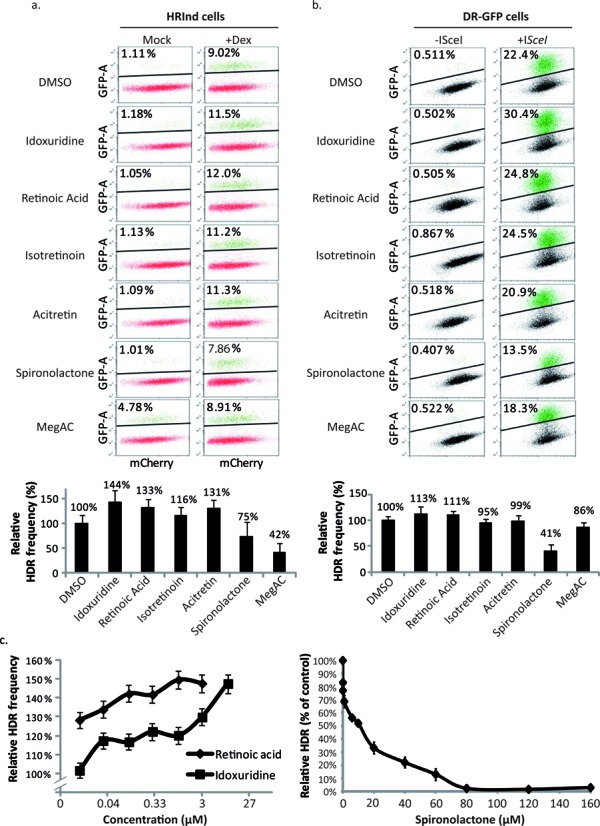
Validation demonstrating that Spiro, RA and Idoxuridine modulate HDR. (**a, b**) Flow cytometry images of cells that were incubated with the indicated compounds and were either mock treated or induced for DSB formation at the DRGFP cassette. Top: representative images of flow cytometry experiments, the percentage indicates GFP-positive cells in the gates. Bottom: quantification of at least three flow cytometry experiments. The relative HDR intensity was calculated relatively to the control (DMSO; bottom). (**a**) HRind cells incubated with EtOh (Mock) or with Dex (Dex) for DSB induction. (**b**) U2OS-DRGFP cells that do not stably express IS*ceI*, infected (+IS*ceI*) or not (−IS*ceI*) with adenovirus expressing IS*ceI*. (**c**) Dose-response curves for validated HDR modulators. Left: HRind cells were incubated with the indicated compounds and concentrations and the fraction of GFP-positive cells was determined as in Figure 1. Shown are average and SEM of three repeats. Right: U2OS-DRGFP cells were infected with an adenovirus that expresses I*SceI* were incubated with different Spiro concentrations and the fraction of GFP-positive cells was determined as in (b).

Next, we validated the effect of the potential candidates identified in our screen on HDR using flow cytometry. We tested RA, Idoxuridine, Acitretin, Isotretinoin, Aripiprazole, MegAc and Spiro, which, reassuringly, all confirmed the results from our screen (Figure 3). In this experiment, different compounds and DMSO were added to HRind cells for an hour followed by a mock treatment or incubation, for two and a half days, with the ligand Dexamethasone (Dex), which induces DSB by translocating mCherry-I*SceI*-GRLBD into the nucleus. The ratio of HDR events following incubation with the different compounds or DMSO was measured (Figure 3a). While growing the HRind cells for flow cytometry analysis, which require plating the cells in a higher density compared to the screen, Aripiprazole and Hycanthone were found to be toxic (data not shown) and hence we excluded them from further analyses. Notably, MegAc repeatedly had a 4-fold higher background of HDR events in the absence of DSB formation (∼4% GFP-positive cells; Figure 3a, MegAc), suggesting that it affects the GR and thus the nuclear location of the endonuclease. To exclude the possibility that the effects seen for the chemical compounds analyzed were due to the GR translocation to the nucleus (31,32), we examined the HDR modulators using an assay, which is independent of the GR. To this end, we infected U2OS-DRGFP cells, which stably contain the DRGFP cassette (33), with an adenovirus expressing the I*SceI* endonuclease (a kind gift from F. Graham (McMaster University, Canada)). As we speculated, the background level of MegAc dropped down to ∼0.5%, which is similar to the levels found in cells treated with DMSO, suggesting that MegAc partially influence the mCherry-I*SceI*-GRLBD chimera even in the absence of inducer. MegAc effect on HDR was significantly reduced (Figure 3b, MegAc), suggesting that it acts as an antagonist in the experimental system (HRind cells) rather than having an effect on HDR, and therefore we excluded it from further analyses. Retinoids can activate the GR (34,35). Indeed, when inducing the DSB with infection of I*SceI*, the effects of the retinoids, Isotretinin and Acitretin, seen with the HRind system, which is an inducible GR-based system, were abolished (Figure 3a, and b), indicating that they are also an artifact of the system. RA, despite being a retinoid, still had an effect of ∼10% increase on HDR in the non-GR system. Idoxuridine, a nucleoside analogue had an increasing effect on HDR frequency of more than 10% following I*SceI* infection (Figure 3). Interestingly, Spiro, despite being a steroid, had the most significant effect when analyzed with a non-GR system, a decrease of more than 50% in HDR frequency (Figure 3). The usage of a non-GR system confirmed that Spiro reduces while RA and Idoxuridine increase HDR frequency. Moreover, dose-response analysis, using either the HRind or the viral I*SceI* system, revealed that all three compounds act in a dose-dependent manner (Figure 3c). Notably, Spiro, at a high concentration, almost withhold HDR of the I*SceI*-induced DSBs (Figure 3c, right) and the IC_50_ was calculated to 15.2 μM (Supplementary Figure S2a).

Next, we examined the effect of the three compounds that passed the validation steps (RA, Spiro and Idoxuridine) on cell cycle progression (Supplementary Figure S3). Idoxuridine, RA and Spiro did not have a significant effect on cell cycle progression (Supplementary Figure S3).

**Figure 4.**
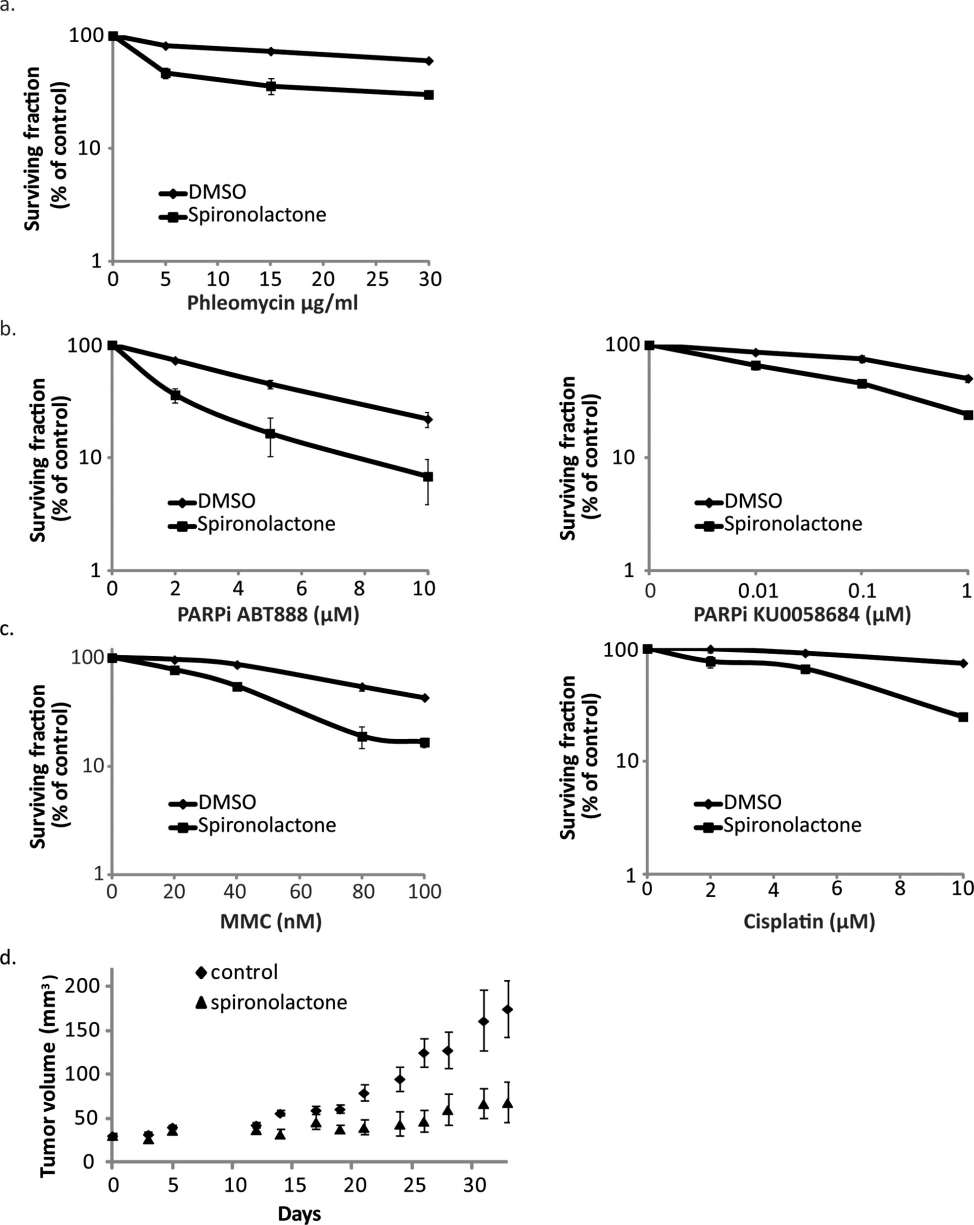
Spironolactone affects cell survival and inhibits tumor growth in mice. (**a**) Clonogenic survival of U2OS cells treated with the Spiro (10 μM), exposed to increasing concentrations of Phleomycin. SD represent the errors from three triplicates of the depicted experiment. (**b**) and (**c**) As in (a), Survival assays of U2OS cells treated with the Spiro (10 μM), exposed to increasing concentrations of PARP inhibitors ABT888 and KU0058684, MMC or Cisplatin. (**d**). HeLa cells were injected into Nod-SCID (1 million HeLa cells suspended in a total volume of 100 μl growing media to each flank). After tumor establishment, mice were treated with Spiro (25 μg per g body weight) or DMSO, three times per week. Tumors were measured three times per week.

Cells that harbor defects in HDR are hypersensitive to various chemotherapy drugs and PARP inhibitors (36,37). Therefore, we hypothesized that Spiro would sensitize human cancer cells to radiomimetic drugs and enhance PARPi-induced lethality. To test this possibility, we assessed cell survival in U2OS cells treated with 10 μM of Spiro that was shown not to affect cell survival when added alone (Supplementary Figure S2b) and with increasing concentrations of Phleomycin (Figure 4a). As expected, when incubating the cells with Spiro in the presence of Phleomycin, cells showed higher sensitivity, with a decrease in colony formation (Figure 4a). Interestingly, combining PARPi with Spiro also results in fewer colonies, as demonstrated with two different PARP inhibitors (Figure 4b). Since HR affects mainly sensitivity in DNA cross-linking agents, we assessed cell survival in cells treated with combination of Spiro and Cisplatin and MMC. Indeed, as depicted in Figure 4c, Spiro addition increased the cell sensitivity of both cross-linking agents (Figure 4c).

Spiro, an FDA approved drug, reduced DSB repair by HDR (Figures 2 and 3) and inhibited cell survival and colony formation of cancer cells in culture (Figure 4a, b, c and Supplementary Figure 2b). Next, we wanted to test whether it can also inhibit tumor growth *in vivo*. To this end, NOD-SCID mice aged 4–8 weeks were subcutaneously injected with 10^6^ HeLa cells suspended in a total volume of 100 μl growing media to each flank. After tumor establishment, mice were treated with Spiro (25 μg per g body weight) or DMSO, three times per week (double blind). Tumors were measured three times per week. The tumor growth of mice treated with Spiro was significantly reduced, with some of the tumors totally obliterated, demonstrating that Spiro may act to inhibit tumor growth *in vivo* (Figure 4d).

**Figure 5.**
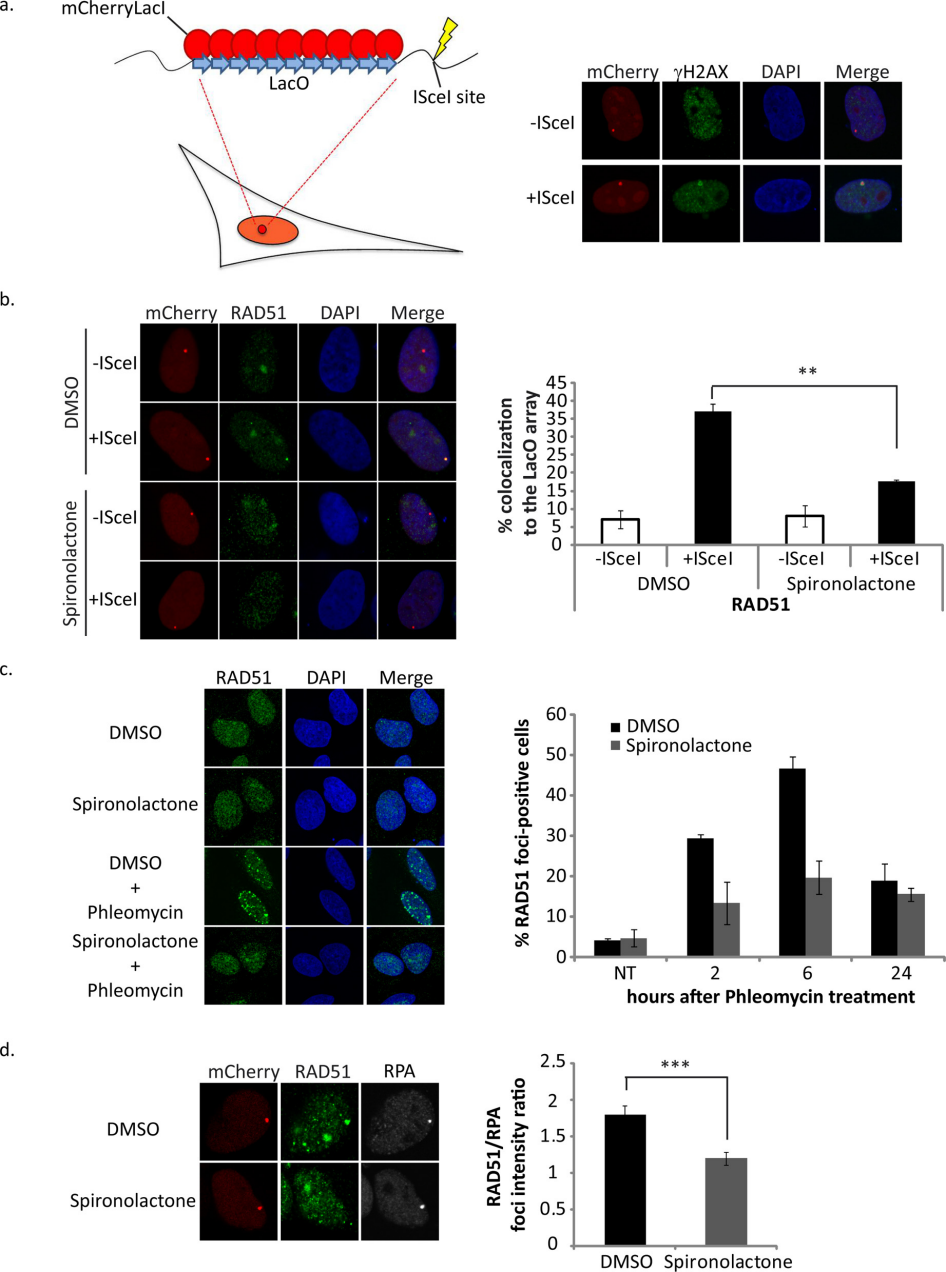
Spironolactone impairs recruitment of HDR related factors. (**a**) Schematic representation of the LacO/I*SceI* system. (**b**) Quantification of RAD51 recruitment on the LacO array after treatment with Spiro. U2OS cells were co-transfected with mCherryLacI and HA-I*SceI* or empty vector and treated at the same time with Spiro (40 μM). After 24 h, cells were fixed and stained for RAD51. The number of co-localization and RAD51 foci with the lacO array were counted in 100 cells. SD represent the errors from three independent experiments. Statistical significance was calculated using the t-test (*p < 0.05, **p < 0.01, ***p < 0.001). (**c**) Spiro affects the kinetics of RAD51 foci formation. U2OS cells were treated with Spiro (40 μM) or DMSO for 24 h followed by treatment of Phleomycin for 1 h and release for 2, 6 and 24 h in medium containing Spiro or DMSO. The cells were subsequently fixed and stained with the RAD51 antibody. The number of foci positive cells (>3) was measured for each time point after analyzing 100 cells for each condition. SD represent the errors from two independent experiments. (**d**) Spiro affects RAD51 foci intensity. U2OS 19 cells were treated as in (b) and finally were fixed and stained with antibodies against RAD51 and RPA. Photos of 100 cells were obtained at a confocal microscope and the focus area intensity was quantified for RAD51 (green) and RPA (cy5) using the Fiji software. The ratio between RAD51 focus intensity to the RPA focus intensity is depicted in the right panel graph. Statistical significance was calculated using the t-test ( **p < 0.01, ***p < 0.001).

To get more insights into the mechanism that leads to the impairment of HR, we asked whether DNA repair proteins involved in HR are properly recruited at DSBs in cells treated with Spiro. To tackle this question, we used a cellular system in which a single DSB can be created at a specific genomic site. This system consists of an array composed of 256 repeats of the lac operator (LacO) flanked by an I*SceI* restriction enzyme site stable integrated in U2OS cells. Visualization of the break is achieved by the expression of lac repressor (LacI) fused to GFP and expression of I*SceI* induces a DSB that is exemplified by the early DNA damage response (DDR) marker γH2AX (Figure 5a, and (38)). Interestingly, the recruitment of BRCA1 and more particularly RAD51 at the LacO array upon DNA damage is impaired in cells treated with Spiro compared to cells treated with DMSO (Figure 5b and Supplementary Figure S4b). Similar decrease in RAD51 foci formation upon DNA damage and in the presence of Spiro was observed when cells were treated with Phleomycin (Figure 5c).

To investigate whether Spiro acts on RAD51 potential to bind single-strand DNA and its strand exchange activity, we performed D-loop formation assay *in vitro* in the presence of Spiro. Our results depicted in Supplementary Figure 5 show that Spiro does not affect RAD51 activity and filament formation *in vitro*. There results, together with the results indicating less foci formation by RAD51 in the presence of Spiro (Figure 5), suggest that Spiro affects the loading of RAD51 in DSBs in cells. To get more insights into the dynamics of the RAD51 focus formation, we performed time course analysis and we checked the kinetics of RAD51 foci formation in Phleomycin and I*SceI* induced breaks. We find that in both cases foci formation is delayed and never reaches the levels of the control (DMSO) (Figure 5c and Supplementary Figure S6). Moreover, when we focus on cells that have RAD51 foci in the Spiro treated cells we find that the focus is less intense (Figure 5d). These results suggest that Spiro affects the loading and the retention of RAD51 *in vivo*.

**Figure 6.**
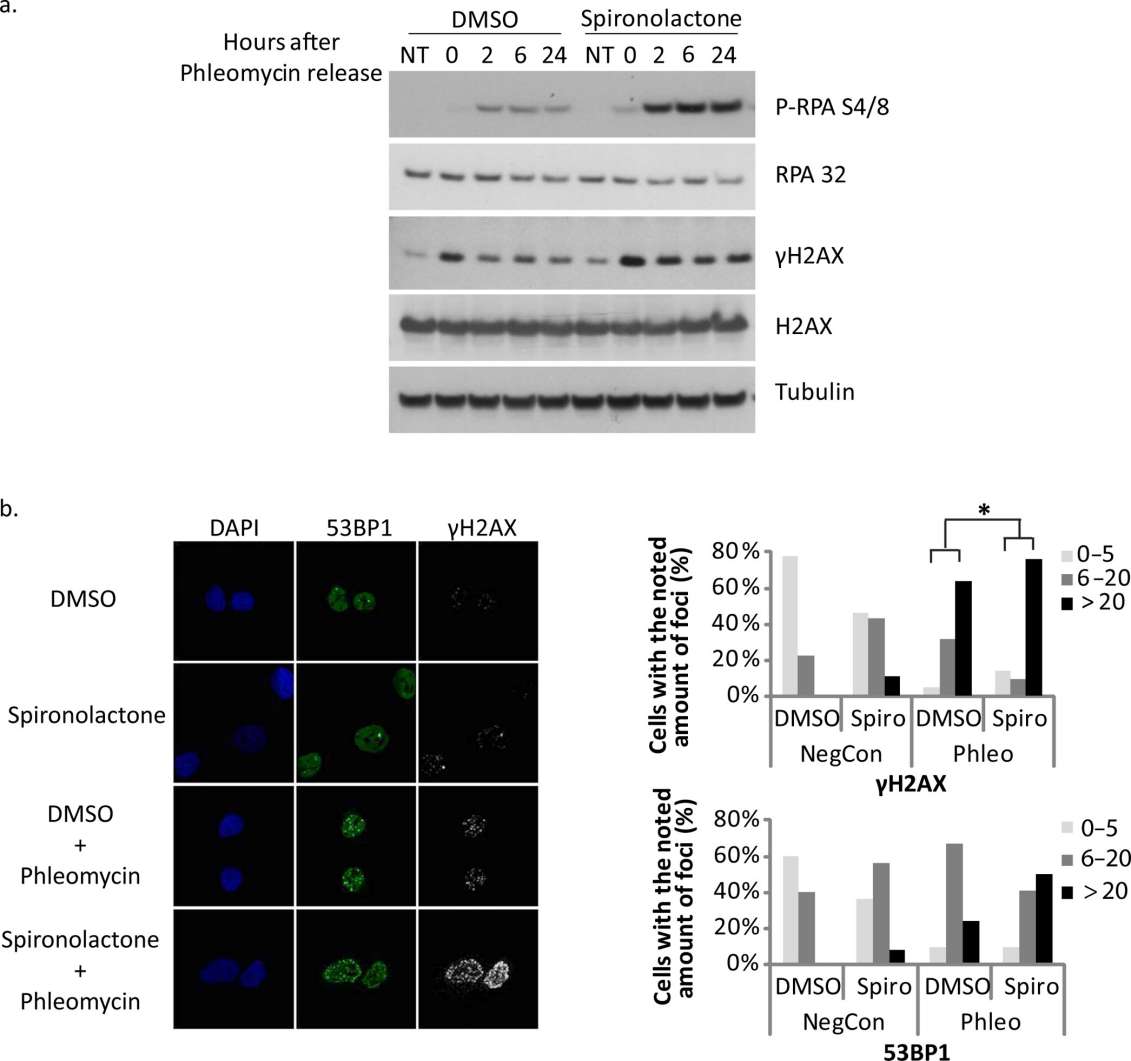
Spironolactone treatment leads to persistent DNA damage. (**a**) U2OS cells were treated with Spiro (40 μM) or DMSO for 24 h followed by treatment of Phleomycin for 1 h and harvested after 0, 2, 6 or 24 h after this treatment. Cells were lyzed in RIPA buffer and analyzed by western blot against the proteins RPA, H2AX and their phosphorylated versions (S4/8 for RPA and S139 for H2AX). Tubulin was used as a loading control. (**b**) Left: U2OS cells were treated with Spiro or DMSO for 1 h, Phleomycin-treated for 1 h and left for recover for 6 h in the presence of Spiro or DMSO. Next, the cells were fixed and co-stained for 53BP1, γH2AX and DAPI. On the right, quantification of foci formation by γH2AX (top) and by 53BP1 (bottom) from two different immunofluorescence experiments. Cells were divided into subgroups according to the amount of foci (0–5, 6–20 or >20) per cell. Statistical significance was calculated using the one tailed heteroscedastic t-test (*p < 0.05).

Spiro treatment did not have a major effect on the protein levels of RAD51 and BRCA1 (Supplementary Figure S4a). Moreover, the impairment of the recruitment of RAD51 is not due to a defect in DNA end resection. Indeed, the percentage of cells with CtIP, an activator of resection, at the LacO upon DNA damage is similar in cells treated with Spiro and DMSO (Supplementary Figure S4b). Interestingly, when we assessed the levels of p-RPA by Western Blot (WB), we observed a significant increase in resection exemplified by increased p-RPA levels (Figure 6a). Similarly, cells treated with Spiro showed increased and sustained levels of γH2AX (Figure 6a). Moreover, analyzing the effect of Spiro on foci formation by γH2AX and 53BP1 due to Phleomycin treatment reveals an increased number of foci per cell in the presence of Spiro (Figure 6b). The above results are indicative of elevated DDR and unrepaired breaks under these conditions.

Altogether, our results indicate that Spiro modulates HDR by affecting the RAD51 foci formation at DSBs.

## DISCUSSION

Radiation therapy and chemotherapy aims to cure cancer by eradicating tumor cells. Both induce a variety of DNA lesions, including oxidative base damages, single-strand breaks and DSBs, which affect the DNA integrity, leading to cell death (39). The use of high-energy radiotherapy and high dose chemotherapy risks the development of side effects and the generation of secondary malignancies in the surrounding normal tissues. A main challenge of radiation therapy nowadays is to maximize specific tumor cell killing and minimize the normal tissue side effects. Thus, the discovery of compounds that confer radiosensitization specifically in cancer cells is of major importance.

Here we have performed a chemical screen to identify compounds that alter HDR. We utilized a library with compounds that are already used in clinic for treatment of other diseases (http://www.prestwickchemical.com/index.php?pa=26). The advantage of such a screen is that the drugs have been tested in humans and the concentrations that the drugs are toxic as well as their side effects are widely known. It therefore obviates the long years of testing novel drugs for safety and stability in the human body, and the drug is ready for doctors’ perception. Moreover, this work provides a significant step in robust high-throughput screens methodology by using an inducible system, which takes an advantage of protein localization control and its live visualization using fluorescent proteins. The provided list of FDA approved small molecules, which modulate HDR to different extents, will be of benefit for the genomic stability community, enabling to further study the mechanisms behind the control of this important repair pathway.

The observation that components of the HDR pathway are mutated or aberrantly expressed in many tumors (36), and the correlation between tumor radio-resistance and increased HDR activity make this repair pathway an attractive therapeutic target. The development of small-molecule inhibitors that can decrease HDR efficiency can be an important strategy to improve radio and chemotherapy treatment of cancer.

Spiro is a synthetic, steroidal agent that acts predominantly as a competitive antagonist of the aldosterone (or mineralocorticoid) receptor (40). It acts by indirect inhibition of sodium reabsorption through the epithelial sodium channel and stimulation of potassium retention, being therefore classified among potassium-sparing diuretics (41). Because of its non-selective binding to Mineralocorticoid receptor it exerts antiandrogen and weak progestogen properties, as well as some indirect estrogen and glucocorticoid effects (32). Spiro is primarily used as a diuretic and antihypertensive, but also for inhibiting androgen activity in the body (40). Here, we describe a novel effect of Spiro on DNA repair by HDR. We also demonstrate that Spiro confers synthetic lethality to radiomimetic drugs and PARP inhibitors and cross-linking agents such as MMC and Cisplatin in cancer cells and inhibits tumor growth in xenografts in mice. Our results suggest Spiro as a new candidate for chemotherapy.

Studies that aimed to investigate whether exposure to Spiro affects the risk of incident breast cancer in women over 55 years of age showed that the long-term management of cardiovascular conditions with Spiro does not increase the risk of breast cancer in women older than 55 years with no history of the disease (42). These results suggest that Spiro action is specific in cancer cells and does not affect normal cells.

During the recent years, several labs have performed chemical screens and identified compounds that inhibit RAD51 filament formation or activity in purified biochemical systems (21,22,23,24). Two of these compounds (B02 and RI-1) were also shown to sensitize cells to DNA damaging agents and PARP inhibitors. In these studies, the compounds were not tested in animal tumor models or tumor xenografts making difficult to conclude whether they could be potent in a clinical setting. Our data show that Spiro treatment in mice xenografts derived from HeLa cells injected subcutaneous decreased the development of the tumor pointing to its potential as a chemotherapy agent. Although we have focused in this study on Spiro, the screen also identified two compounds that augment HDR efficiency; these molecules may be utilized in gene targeting. The three chemical compounds that we identified as modifiers of HDR may act at different stages of the HDR pathway. This is a benefit of our screen methodology that is based on the HRind system, which relies on *in vivo* HDR events and not on *in vitro* studies focused on one HR factor. Revealing the mode of action of the different compounds during HDR is important for our understanding of this repair pathway.

Reported chemicals that affect HR via inhibition of RAD51 activity or filament formation (21,22,23,24) are all aromatic molecules while Spiro is a steroid suggesting different modes of action of these compounds on RAD51 foci formation. Indeed, our *in vitro* assays show no effect of Spiro in D-loop formation. On the other hand, Spiro affects RAD51 foci formation in cells, suggesting that loading or retention of RAD51 at the sites of damage is impaired. Spiro reduces HDR by affecting the RAD51 foci formation, yet HDR may be affected at different steps of the HDR pathway. The discovery that Spiro does not directly affect the activity of RAD51 (at least *in vitro*) highlights the power of the screen we have implemented, which scored for novel compounds that alter HDR in cells and can affect different components in this process. This is in contrast to being limited directed to examine activities of specific proteins, therefore missing compounds that affect other, even unknown compounds of the HDR machinery. Therefore, beyond the discovery of Spiro as a putative drug for chemotherapy, we provide a resource for chemicals that alter HDR to different extents and hope it will serve the community in shedding light on the DDR.

Although further testing in xenografts, using different cell types other than HeLa, as well as in a variety of solid tumors will be necessary to evaluate the full potential of Spiro as chemotherapeutic agent, this work presents an important step towards the development of new drugs in cancer therapy.

## SUPPLEMENTARY DATA

Supplementary Data are available at NAR Online.

SUPPLEMENTARY DATA
