# Supplementary material for: A high-throughput chemical screen with FDA approved drugs reveals that the antihypertensive drug Spironolactone impairs cancer cell survival by inhibiting homology directed repair
Source: Nucleic Acids Res. 2014 Mar 25;42(9):5689–701. doi: 10.1093/nar/gku217 (PMC4027216; doi:10.1093/nar/gku217)
Supplement: SUPPLEMENTARY DATA [file supp_42_9_5689__index.html]

A high-throughput chemical screen with FDA approved drugs reveals that the antihypertensive drug Spironolactone impairs cancer cell survival by inhibiting homology directed repair — SUPPLEMENTARY DATA 

# A high-throughput chemical screen with FDA approved drugs reveals that the antihypertensive drug Spironolactone impairs cancer cell survival by inhibiting homology directed repair

## SUPPLEMENTARY DATA

**Files in this Data Supplement:**

- SUPPLEMENTARY DATA
