# Supplementary material for: A high-throughput chemical screen with FDA approved drugs reveals that the antihypertensive drug Spironolactone impairs cancer cell survival by inhibiting homology directed repair
Source: Nucleic Acids Res. 2014 Mar 25;42(9):5689–701. doi: 10.1093/nar/gku217 (PMC4027216; doi:10.1093/nar/gku217)
Supplement: SUPPLEMENTARY DATA [file supp_gku217_nar-03572-f-2013-File002.pdf]

Supplementary figure legends:

Supplementary Figure S1. Chemical structures of similar compounds found in the screen. (a) The retinoids, Retinoic acid, Acitretin and Isotretinoin. (b) The steroids, Spironolactone and Megesterol acetate.

Supplementary Figure S2. (a) IC_{50} calculation for Spiro on HDR efficiency. (b) Survival assay of U2OS cells using the indicated amounts of Spiro. Average and s.d. of 2 independent experiments is shown.

Supplementary Figure S3. Cells were incubated with Hoechst for 15 min and analyzed for DNA content using flow cytometry. (a) Cell cycle profile of HRind cells incubated for 24 hours with the different chemicals, DMSO as mock, or nocodazole (16 hours) as a control for cell cycle perturbation. (b) Quantification of 5 independent repeats as in (a). Statistical analysis of the G1 populations in different treatments was performed using t-test and only nocodazole treatment showed a significant change of the population of G1 cells (*).

Supplementary Figure S4. (a) BRCA1 and RAD51 protein levels after Phleomycin and Spiro treatment (as in Figure 6a). Western blots were quantified using the ImageJ software and the relative protein levels of RAD51 and BRCA1 were calculated as a ratio to the tubulin levels. s.d. represent the errors from 2 independent experiments. (b) Quantification of CtIP and BRCA1 recruitment on the LacO array after treatment with Spiro (as in Figure 5b) (43).

Supplementary Figure S5. Effect of Spironolactone on the DNA strand exchange activity of RAD51. (a) Experimental scheme of the D-loop assay. The asterisk denotes the ^{32}P label on the 5′-ssDNA end. (b) Effect of the Spironolactone concentrations on the RAD51 (0.3 μM) DNA strand exchange activity. D-loop formation by RAD51

between ^{32}P-labeled 90-mer ssDNA (oligo#90) (0.9 μM nt) and pUC19 supercoiled dsDNA (15 μM, nt) was analyzed by electrophoresis in a 1% agarose gel. (c) The results were plotted as a graph. The yield of D-loop formation in the absence of Spironolactone was expressed as 100% (the actual yield expressed as a percentage of the total plasmid DNA was 31.6 %). Control containing no RAD51 is shown in lane 1. The error bars represent standard error of the mean (S.E.M).

Supplementary Figure S6. Time course of RAD51 recruitment to the LacO array. U2OS 19 GFP Lac cells (43) that are inducible with Doxycycline for the expression of I*SceI,* were grown on coverslips and treated with Spiro (40 μM) and Doxycycline (1 μg/ml) for the indicated time points. The cells were fixed and stained with an antibody against RAD51. The number of co-localization between the array and RAD51 foci were counted in 100 cells. s.d. represent the errors from 2 independent experiments.

Supplementary table legend

Supplementary Table S1. List of the different compounds and controls incubated with TA and the frequency of GFP positive cells obtained in the HDR screen. Compounds in which the cell count was lower than 2500 or the signal was not specific or not validated while repeating the experiment were omitted. Green and red indicate more than 3 or 4 s.d., respectively.

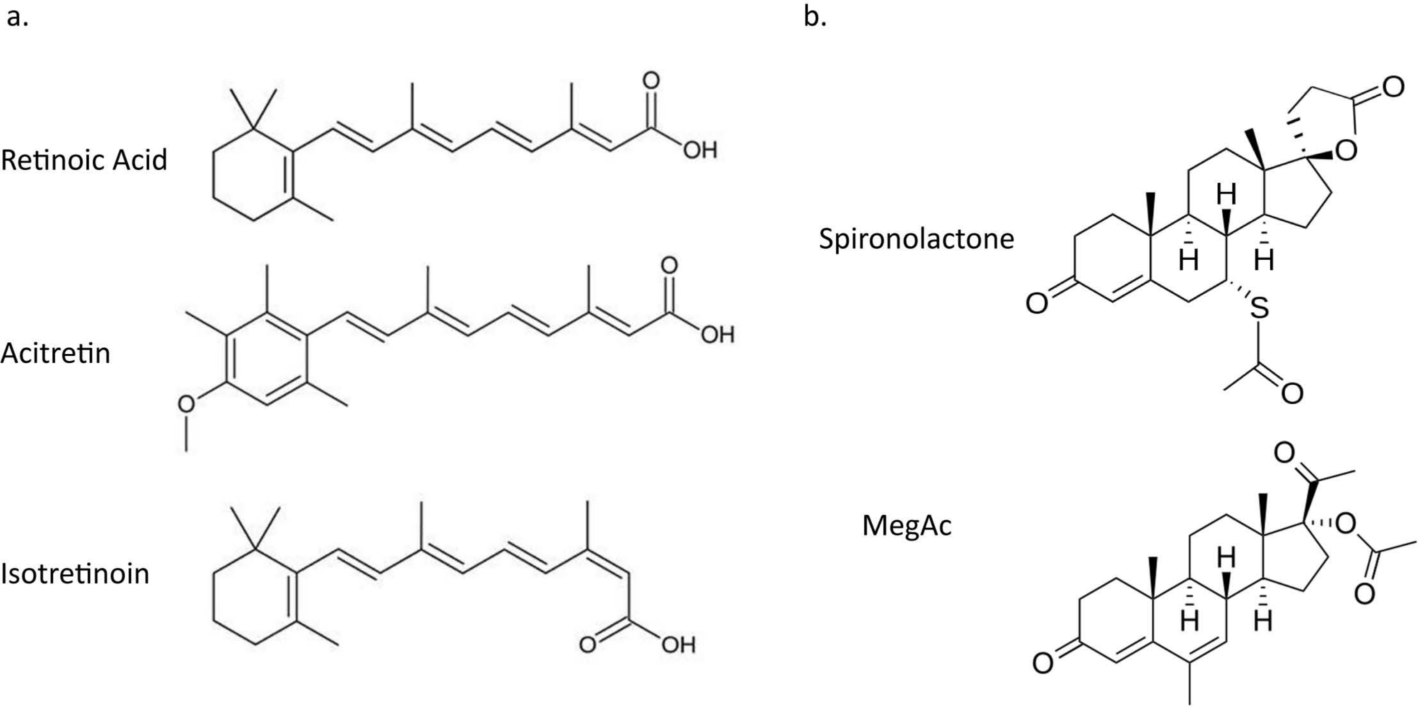

Supplementary Figure S1.

a.

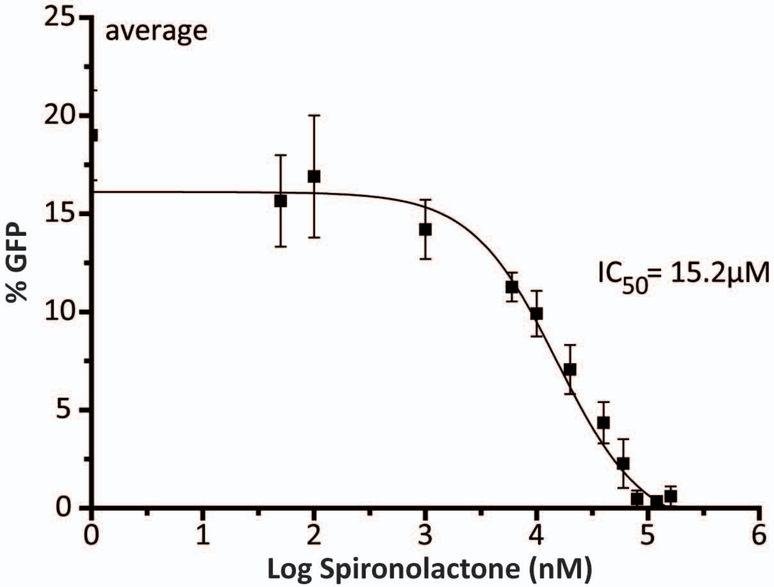

Model	DoseResp		
Equation	y = A1 + (A2-A1)/(1 + 10^((LOGx0-x) *p))		
Reduced Chi-Sqr	1.07237		
Adj. R-Square	0.97642		
		Value	Standard Error
Mean	A1	-1.20123	1.05761
Mean	A2	16.10729	1.26414
Mean	LOGx0	4.18402	0.09925
Mean	p	-1.12226	0.25452
Mean	span	17.30852	1.90895
Mean	EC20	52540.68879	20697.12776
Mean	EC50	15276.45446	3490.99185
Mean	EC80	4441.70159	1447.72584

b.

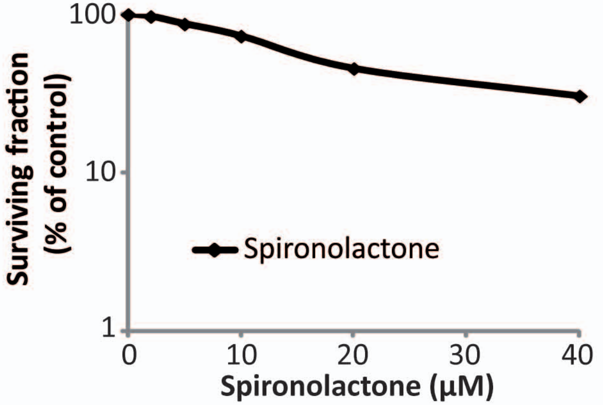

Supplementary Figure S2.

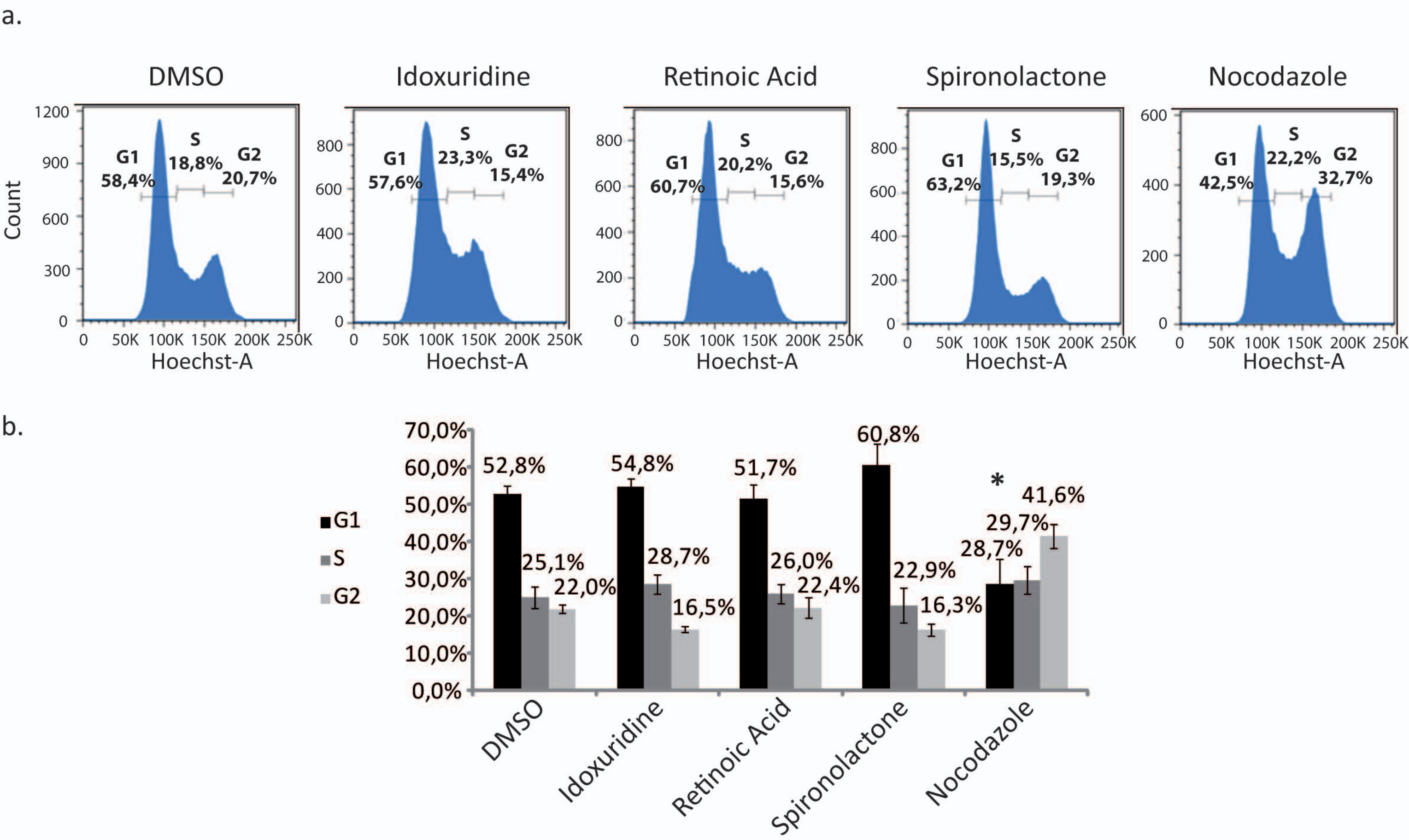

Supplementary Figure S3.

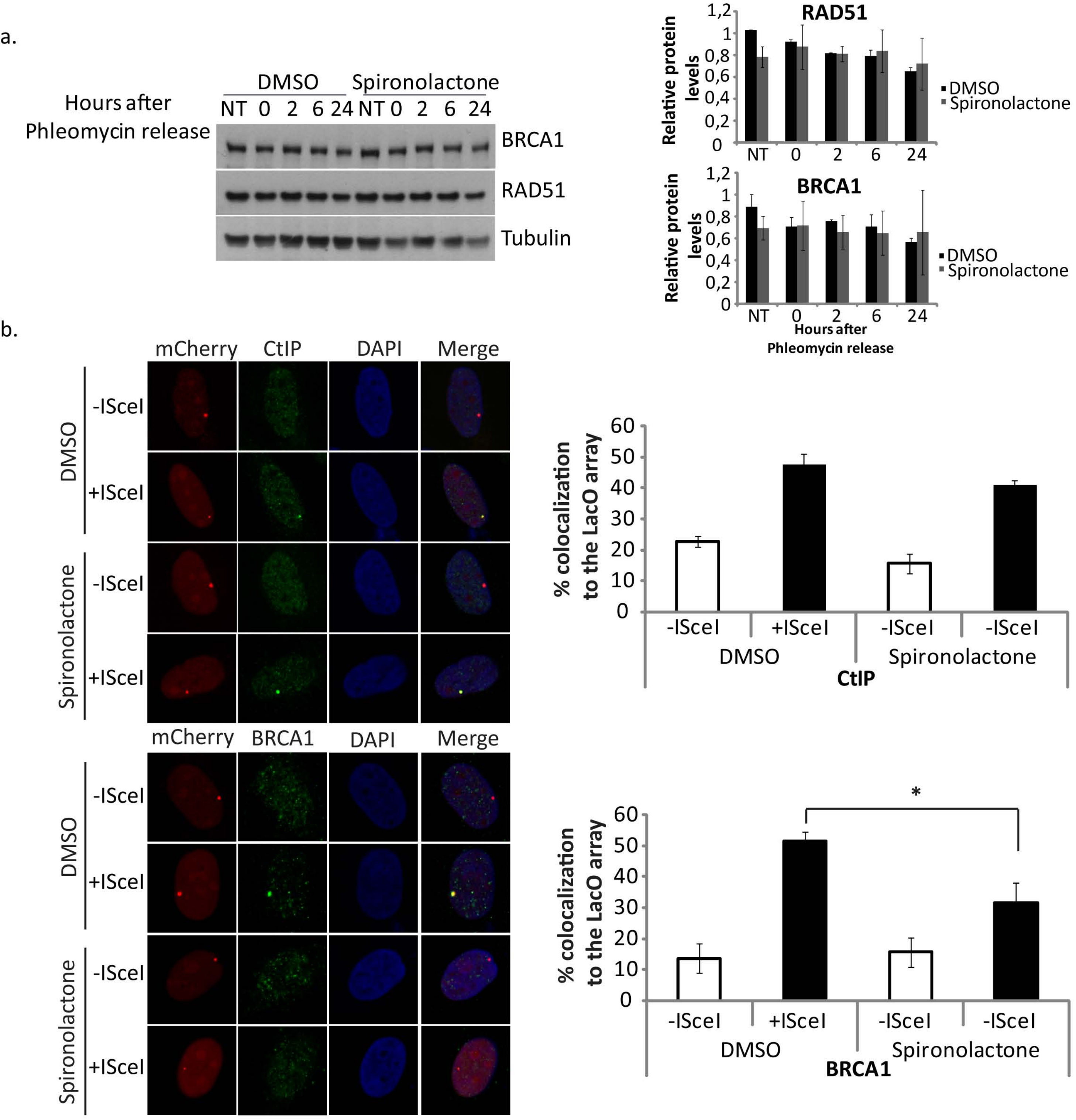

Supplementary Figure S4.

a.

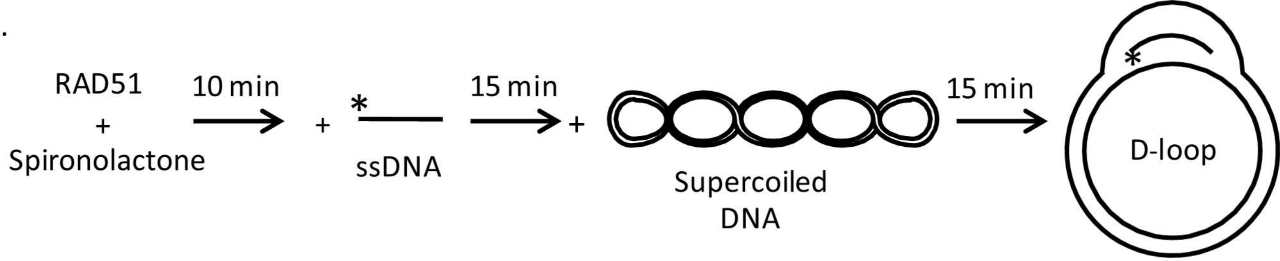

b.

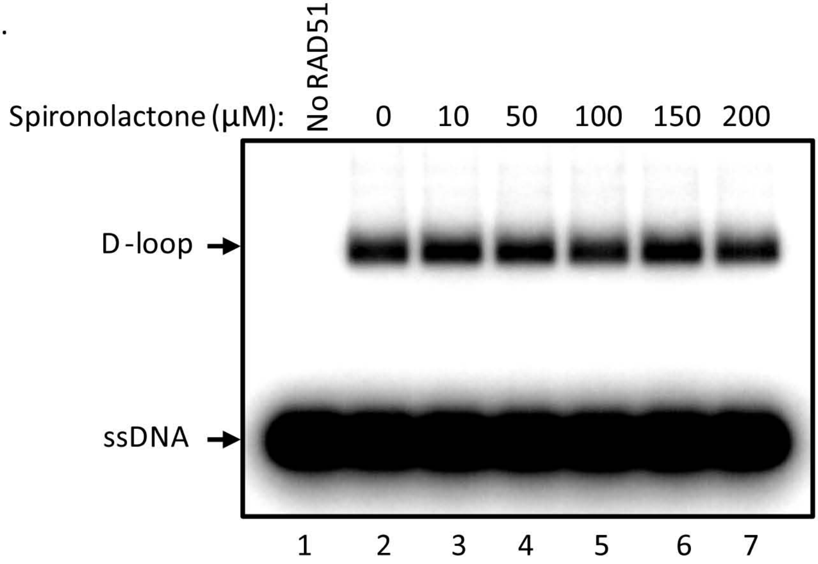

c.

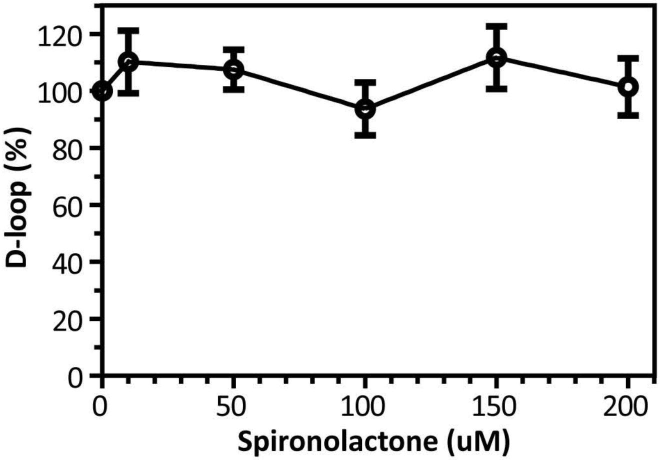

Supplementery Figure S5.

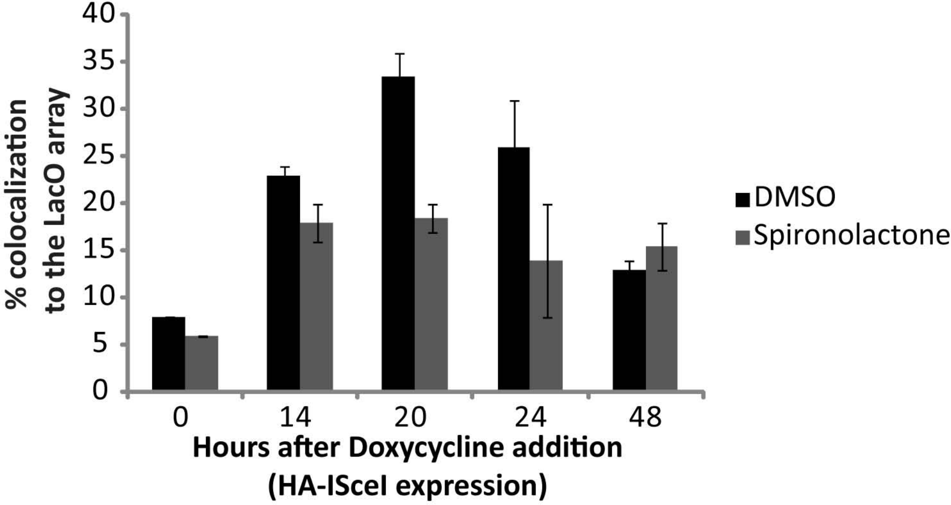

Supplementary Figure S6.

Supplementary Table 1

Thresholds:

<3SD	>3SD	<4SD	>4SD
7,7%	11,5%	7,1%	12,1%

Drugs	% of positive cells Mean	SD	Cell count Mean	SD
No inhibitor+TA	9,57%	0,63%	5009	341
DNAPK inhibitor 30µM	14,25%	0,72%	2547	233
ATM inhibitor 10µM	6,70%	0,61%	3981	270
**Drugs**	Mean	SEM	Mean	SD
Retinoic acid	12,81%	0,48%	4548	367
Deflazacort	12,65%	0,47%	4788	214
Acitretin	12,45%	0,46%	5096	64
Isotretinoin	12,32%	0,45%	4806	661
Idoxuridine	12,30%	0,44%	5580	98
Escitalopram	12,00%	0,45%	5210	103
Niflumic acid	11,82%	0,46%	4184	905
Pronethalol hydrochloride	11,78%	0,43%	5400	239
Pindolol	11,75%	0,45%	4954	118
Canrenoic acid potassium salt	11,69%	0,46%	4830	198
Dehydrocholic acid	11,66%	0,44%	5314	148
Estradiol-17 beta	11,63%	0,49%	4359	NA
Acebutolol hydrochloride	11,62%	0,42%	5762	172
Iproniazide phosphate	11,61%	0,44%	5092	192
Trazodone hydrochloride	11,58%	0,49%	3318	1265
Azaperone	11,54%	0,47%	4306	403
Procainamide hydrochloride	11,51%	0,44%	5078	326
Spiramycin	11,46%	0,44%	4378	1173
Hydrocortisone base	11,45%	0,44%	5203	NA
Flumethasone	11,45%	0,43%	4394	1468
Dapsone	11,44%	0,43%	5446	170
Gabazine bromide	11,43%	0,45%	4941	NA
Disulfiram	11,39%	0,57%	2696	522
Cimetidine	11,35%	0,43%	5360	121
Metrizamide	11,32%	0,46%	4725	NA
Aztreonam	11,32%	0,45%	4896	12
Benzamil hydrochloride	11,29%	0,44%	4989	133
Fenofibrate	11,28%	0,43%	5342	133
Guaifenesin	11,25%	0,44%	4220	1496
Nicotinamide	11,24%	0,44%	4774	494
Naltrexone hydrochloride dihydrate	11,23%	0,44%	5207	16
Azacyclonol	11,19%	0,45%	4800	9
Telenzepine dihydrochloride	11,19%	0,51%	3166	1021
Dibenzepine hydrochloride	11,19%	0,46%	4648	NA
Bambuterol hydrochloride	11,18%	0,43%	4580	961
Zardaverine	11,18%	0,43%	5132	421
Edrophonium chloride	11,18%	0,45%	4264	802
Diltiazem hydrochloride	11,18%	0,42%	5408	133
Carbarsone	11,15%	0,49%	4079	NA
Rosiglitazone Hydrochloride	11,15%	0,45%	4526	650
Paromomycin sulfate	11,15%	0,44%	4922	320
Procyclidine hydrochloride	11,12%	0,44%	5058	159
Adamantamine fumarate	11,11%	0,53%	3547	NA
Dropropizine (R,S)	11,10%	0,42%	5050	743
Chlortetracycline hydrochloride	11,07%	0,42%	5454	260
SR-95639A dihydrochloride	11,07%	0,49%	4157	NA
Chlorpheniramine maleate	11,06%	0,44%	5009	167
Atractyloside potassium salt	11,04%	0,45%	4074	1090
Oxprenolol hydrochloride	11,04%	0,44%	5016	69
Homochlorcyclizine dihydrochloride	11,02%	0,43%	4866	483
Diazoxide	11,02%	0,43%	5254	233
Iopromide	11,01%	0,45%	4536	340
Khellin	11,00%	0,44%	4570	658
Tripelennamine hydrochloride	11,00%	0,43%	5056	346
Trichlorfon	10,99%	0,43%	4988	436
Pramipexole	10,98%	0,41%	5252	663
Butacaine	10,96%	0,43%	5280	145
Bacampicillin hydrochloride	10,95%	0,46%	4519	NA
Levodopa	10,95%	0,43%	5287	107
Nifenazone	10,94%	0,41%	5584	309
Thiamphenicol	10,92%	0,45%	4822	22
Clobetasol propionate	10,91%	0,44%	3854	1522
Dihydroergotamine tartrate	10,91%	0,43%	5026	309
Ketanserin tartrate hydrate	10,89%	0,53%	3173	445
Praziquantel	10,89%	0,43%	4814	463
Sulfathiazole	10,89%	0,42%	5528	76
Lacidipine	10,87%	0,47%	4003	430
Mephenesin	10,86%	0,44%	4952	69
Cephalexin monohydrate	10,84%	0,42%	5078	578
Isoxicam	10,84%	0,41%	5518	361
Dicyclomine hydrochloride	10,84%	0,42%	5356	43
Eserine sulfate, physostigmine sulfate	10,83%	0,45%	3966	1220
Phenformin hydrochloride	10,83%	0,45%	4724	71
Trapidil	10,82%	0,42%	5194	301
Ketotifen fumarate	10,82%	0,41%	4973	942
Mephentermine hemisulfate	10,81%	0,42%	4406	1341
Doxylamine succinate	10,79%	0,41%	5616	122
Itopride	10,78%	0,47%	4389	NA
Chlorotrianisene	10,76%	0,44%	4211	1078
Picotamide monohydrate	10,75%	0,41%	5619	197
Methyldopa (L,-)	10,75%	0,44%	4223	1030
Methazolamide	10,75%	0,42%	5296	64
Trimethobenzamide hydrochloride	10,74%	0,42%	5373	62
Dimenhydrinate	10,73%	0,43%	5003	188
Chlorthalidone	10,72%	0,43%	4586	938
Metaraminol bitartrate	10,72%	0,44%	4914	134
Alclometasone dipropionate	10,72%	0,45%	4160	871
Homatropine hydrobromide (R,S)	10,71%	0,42%	5396	196
Sulfapyridine	10,70%	0,45%	4084	924
Roxithromycin	10,70%	0,44%	4727	177
Neomycin sulfate	10,69%	0,41%	5332	303
Aminophylline	10,68%	0,44%	4922	46
Mevalonic-D, L acid lactone	10,67%	0,44%	4256	1097
Moxonidine	10,67%	0,43%	5136	185
Fludrocortisone acetate	10,67%	0,41%	5074	699
Oxcarbazepine	10,66%	0,43%	5037	NA
Oxybutynin chloride	10,66%	0,43%	5110	30
Bupropion hydrochloride	10,66%	0,42%	4748	969
Isoxsuprine hydrochloride	10,65%	0,44%	4776	255
Labetalol hydrochloride	10,65%	0,44%	4980	46
Pipemidic acid	10,65%	0,43%	5067	153
Cyanocobalamin	10,65%	0,45%	4790	NA
Butoconazole nitrate	10,64%	0,48%	4068	NA
Folinic acid calcium salt	10,64%	0,44%	4202	1152
Chenodiol	10,63%	0,43%	5095	112
Linezolid	10,63%	0,43%	5025	NA
Tetrahydroxy-1,4-quinone monohydrate	10,62%	0,42%	5164	141
Chlorzoxazone	10,62%	0,42%	4458	1183
Ramipril	10,61%	0,46%	4492	7

CONTROLS:

No inhibitor+TA

Plate #	GFP positive cells: % of positive cells Mean	SEM	Cell count Mean	SD
1	10,12%	0,29%	5356	313
2	10,35%	0,30%	5114	120
3	9,54%	0,29%	5150	128
4	10,12%	0,29%	5054	291
5	9,86%	0,30%	4626	313
6	9,89%	0,29%	5154	59
7	8,94%	0,28%	5181	93
8	9,76%	0,30%	4982	40
9	8,82%	0,28%	5190	267
10	9,73%	0,30%	4008	1079
11	10,62%	0,31%	4826	124
12	8,45%	0,27%	5296	111
13	9,06%	0,27%	5334	274
14	8,89%	0,28%	5004	96
15	9,41%	0,28%	4854	538
	**9,57%**	**0,63%**	**5009**	**341**

DNAPK inhibitor 30µM

Plate #	GFP positive cells: % of positive cells Mean	SEM	Cell count Mean	SD
1	15,29%	0,47%	2880	114
2	15,09%	0,49%	2618	263
3	14,49%	0,48%	2660	135
4	14,30%	0,48%	2613	195
5	14,62%	0,49%	2424	209
6	14,08%	0,48%	2627	6
7	13,84%	0,47%	2640	55
8	13,56%	0,49%	2398	197
9	13,89%	0,48%	2571	202
10	14,41%	0,52%	1815	507
11	15,83%	0,51%	2485	37
12	13,21%	0,46%	2642	177
13	13,87%	0,46%	2689	143
14	13,44%	0,47%	2606	168
15	13,88%	0,47%	2537	191
	**14,25%**	**0,72%**	**2547**	**233**

ATM inhibitor 10µM

Plate #	GFP positive cells: % of positive cells Mean	SEM	Cell count Mean	SD
1	7,24%	0,28%	4302	114
2	7,32%	0,29%	4075	196
3	6,63%	0,27%	4091	122
4	7,17%	0,29%	3872	85
5	6,54%	0,27%	3978	295
6	7,42%	0,30%	3860	12
7	6,41%	0,28%	3886	96
8	6,54%	0,28%	3935	153
9	7,26%	0,29%	3997	189
10	6,50%	0,29%	3182	447
11	7,61%	0,29%	4104	53
12	5,63%	0,25%	4057	135
13	6,30%	0,25%	4424	317
14	5,86%	0,27%	3927	101
15	6,05%	0,26%	4021	192
	**6,70%**	**0,61%**	**3981**	**270**

Disopyramide	10,61%	0,43%	5048	235
Acyclovir	10,60%	0,44%	4786	196
Acarbose	10,60%	0,44%	4571	528
Cefsulodin sodium salt	10,60%	0,43%	5002	132
Trimeprazine tartrate	10,59%	0,45%	4681	55
Meclofenoxate hydrochloride	10,59%	0,43%	4386	1207
Cefdinir	10,58%	0,41%	5178	552
Tizanidine HCl	10,55%	0,43%	4724	477
Alprenolol hydrochloride	10,55%	0,43%	4448	1069
Nifurtimox	10,55%	0,44%	4680	152
Tranylcypromine hydrochloride	10,54%	0,44%	4941	0
Pefloxacine	10,54%	0,45%	4639	NA
Practolol	10,54%	0,43%	4570	770
Fenspiride hydrochloride	10,54%	0,43%	3842	1854
Betaxolol hydrochloride	10,54%	0,53%	3090	382
Tolazoline hydrochloride	10,53%	0,40%	5802	120
Nafcillin sodium salt monohydrate	10,53%	0,41%	5207	402
Indapamide	10,52%	0,42%	5384	71
Azlocillin sodium salt	10,52%	0,42%	5122	474
Molindone hydrochloride	10,52%	0,46%	4192	231
Nifedipine	10,51%	0,44%	4588	385
Bupivacaine hydrochloride	10,51%	0,42%	5290	39
Chloropyramine hydrochloride	10,51%	0,44%	4805	161
Benzathine benzylpenicillin	10,49%	0,43%	5099	13
Voriconazole	10,49%	0,43%	5004	107
Sulpiride	10,49%	0,41%	5512	65
Sibutramine HCl	10,48%	0,43%	4826	368
5-fluorouracil	10,47%	0,56%	2766	356
Bretylium tosylate	10,47%	0,57%	2574	494
Pranoprofen	10,47%	0,41%	5388	95
Trichlormethiazide	10,46%	0,44%	4802	189
Prednisolone	10,45%	0,41%	5487	197
Ethamivan	10,45%	0,45%	4632	94
Tinidazole	10,45%	0,43%	4169	1263
Betamethasone	10,44%	0,42%	4729	949
Pargyline hydrochloride	10,44%	0,43%	5092	31
Perindopril	10,43%	0,43%	4937	11
Nomifensine maleate	10,43%	0,41%	5330	477
Naphazoline hydrochloride	10,43%	0,40%	5626	276
Cefadroxil	10,42%	0,42%	5267	NA
Meropenem	10,42%	0,45%	4543	48
Acetohexamide	10,41%	0,41%	5500	43
Troleandomycin	10,41%	0,41%	5244	356
Acetaminophen	10,41%	0,43%	4886	170
Alfaxalone	10,40%	0,41%	5232	376
Levonordefrin	10,40%	0,45%	3727	1188
Ronidazole	10,38%	0,47%	3742	658
Antipyrine, 4-hydroxy	10,37%	0,40%	5596	187
Sulfaguanidine	10,37%	0,42%	5253	182
Orphenadrine hydrochloride	10,37%	0,43%	4954	252
Metolazone	10,36%	0,43%	4938	71
Methylhydantoin-5-(D)	10,36%	0,44%	4616	187
Modafinil	10,36%	0,45%	4585	NA
Tosufloxacin hydrochloride	10,36%	0,43%	5069	NA
Minoxidil	10,35%	0,40%	5560	159
Ampyrone	10,35%	0,41%	4923	775
Zonisamide	10,34%	0,42%	5226	6
Ethionamide	10,34%	0,42%	5212	163
Diphenhydramine hydrochloride	10,34%	0,41%	5516	160
Xylazine	10,34%	0,42%	5148	148
Beclomethasone dipropionate	10,34%	0,43%	5006	62
Cefamandole sodium salt	10,33%	0,42%	4274	1375
Ethotoin	10,33%	0,41%	5388	268
Heptaminol hydrochloride	10,33%	0,41%	5374	219
Clidinium bromide	10,33%	0,42%	5154	25
Mitotane	10,33%	0,43%	4856	309
Ribavirin	10,32%	0,40%	5546	432
Antazoline hydrochloride	10,32%	0,44%	4402	598
Isoflupredone acetate	10,32%	0,40%	5599	99
Spectinomycin dihydrochloride	10,32%	0,42%	5248	47
Moclobemide	10,32%	0,41%	4871	856
Ciprofibrate	10,32%	0,44%	4662	233
Misoprostol	10,31%	0,42%	5112	162
Pentobarbital	10,31%	0,42%	4491	1000
Iocetamic acid	10,31%	0,44%	4778	115
Cortisone	10,31%	0,41%	5353	153
Isosorbide mononitrate	10,31%	0,43%	4958	13
Piromidic acid	10,30%	0,43%	5044	18
Oxalamine citrate salt	10,30%	0,44%	4610	124
Nalbuphine hydrochloride	10,30%	0,41%	5434	140
Entacapone	10,30%	0,42%	5206	93
Erythromycin	10,30%	0,43%	5026	101
Amphotericin B	10,29%	0,46%	4284	NA
Tamoxifen citrate	10,29%	0,44%	4508	233
Procaine hydrochloride	10,29%	0,41%	5313	140
Bethanechol chloride	10,29%	0,43%	4777	460
Meclozine dihydrochloride	10,28%	0,44%	4745	NA
Aceclofenac	10,27%	0,42%	5086	130
Dienestrol	10,27%	0,44%	3760	1552
Articaine hydrochloride	10,27%	0,43%	4788	320
Gemfibrozil	10,27%	0,40%	5686	4
Ascorbic acid	10,27%	0,42%	4472	1059
Tylosin	10,26%	0,42%	5286	6
Isopyrin hydrochloride	10,26%	0,42%	4486	1153
Racecadotril	10,26%	0,41%	4949	625
Flavoxate hydrochloride	10,26%	0,41%	4555	1293
Pyrantel tartrate	10,26%	0,41%	5146	361
Baclofen (R,S)	10,25%	0,43%	4336	1059
Etifenin	10,24%	0,42%	5070	216
Piperacillin sodium salt	10,24%	0,43%	4233	1000
Pyrilamine maleate	10,24%	0,41%	5360	332
Benoxinate hydrochloride	10,24%	0,41%	5580	23
Tropicamide	10,24%	0,41%	5410	180
Olanzapine	10,24%	0,43%	4980	98
Diclofenac sodium	10,24%	0,41%	5344	261
Demeclocycline hydrochloride	10,24%	0,47%	3823	479
Demecarium bromide	10,24%	0,42%	5240	50
Ethacrynic acid	10,23%	0,44%	4314	462
Propantheline bromide	10,23%	0,43%	5018	89
Rifampicin	10,23%	0,43%	4980	76
Pentoxifylline	10,23%	0,41%	5198	252
Hydroxyzine dihydrochloride	10,23%	0,40%	5548	313
Spiperone	10,22%	0,43%	4836	231
Dimethadione	10,22%	0,44%	4751	8
Liothyronine	10,21%	0,44%	4686	157
Lidocaine hydrochloride	10,21%	0,39%	5808	191
Trimipramine maleate salt	10,21%	0,44%	4784	23
Tiaprofenic acid	10,21%	0,43%	4724	288
Tolnaftate	10,21%	0,40%	5586	165
Moxisylyte hydrochoride	10,21%	0,40%	5606	108

Metoprolol-(+,-) (+)-tartrate salt	10,21%	0,43%	4092	1309
Carbamazepine	10,20%	0,41%	5360	251
Iobenguane sulfate	10,20%	0,46%	3892	569
Acefylline	10,20%	0,41%	5322	113
Prilocaine hydrochloride	10,19%	0,42%	5201	122
N6-methyladenosine	10,19%	0,41%	5003	795
Deferoxamine mesylate	10,18%	0,49%	3403	604
Phentolamine hydrochloride	10,18%	0,40%	5540	81
Nicorandil	10,18%	0,42%	5076	310
Letrozole	10,18%	0,44%	4530	235
Metformin hydrochloride	10,18%	0,41%	5284	72
Guanadrel sulfate	10,17%	0,42%	4278	1126
Proguanil hydrochloride	10,17%	0,41%	5027	443
Rimantadine Hydrochloride	10,17%	0,41%	5326	145
Dipyrone	10,17%	0,44%	4524	278
Amfepramone hydrochloride	10,17%	0,42%	5196	93
Reserpine	10,17%	0,46%	3361	1243
Pivmecillinam hydrochloride	10,16%	0,42%	4886	285
Didanosine	10,16%	0,40%	5472	153
Tolmetin sodium salt dihydrate	10,16%	0,41%	5169	208
Betazole hydrochloride	10,16%	0,40%	5554	67
Acetopromazine maleate salt	10,16%	0,43%	4599	639
Streptozotocin	10,16%	0,42%	4102	1440
Idebenone	10,15%	0,50%	3683	NA
Ticlopidine hydrochloride	10,15%	0,40%	5582	129
Bicalutamide	10,15%	0,41%	5166	275
Phenylpropanolamine hydrochloride	10,14%	0,42%	4398	1032
Alizapride HCl	10,14%	0,43%	4970	NA
Clioquinol	10,14%	0,44%	4676	25
Etilefrine hydrochloride	10,14%	0,41%	5162	190
Benzocaine	10,14%	0,43%	4812	44
Fenipentol	10,14%	0,42%	5090	111
Benperidol	10,13%	0,43%	4924	145
Bezafibrate	10,12%	0,39%	5346	711
Ebselen	10,12%	0,42%	4294	1068
Norethynodrel	10,12%	0,40%	5530	104
Racepinephrine HCl	10,12%	0,43%	4606	462
Amoxicillin	10,11%	0,40%	5383	385
Triclosan	10,11%	0,45%	4320	226
Carbimazole	10,11%	0,41%	5458	NA
Ozagrel hydrochloride	10,11%	0,41%	5266	174
Ethynylestradiol 3-methyl ether	10,11%	0,42%	4987	150
Cromolyn disodium salt	10,11%	0,43%	4828	111
Zomepirac sodium salt	10,11%	0,43%	4090	1043
Liranaftate	10,11%	0,44%	3936	1177
Enoxacin	10,11%	0,42%	5060	176
Glipizide	10,10%	0,42%	4655	752
Norethindrone	10,10%	0,42%	4586	921
Thioproperazine dimesylate	10,10%	0,41%	5224	214
Ifenprodil tartrate	10,10%	0,41%	5198	350
Pirenzepine dihydrochloride	10,10%	0,41%	5334	158
Tracazolate hydrochloride	10,10%	0,41%	5092	334
Carisoprodol	10,10%	0,42%	5032	NA
Asenapine maleate	10,09%	0,42%	5016	233
Enalapril maleate	10,09%	0,41%	5348	170
Butirosin disulfate salt	10,09%	0,40%	5382	319
Viloxazine hydrochloride	10,09%	0,42%	5162	60
Pinacidil	10,08%	0,43%	4146	1190
Trimethadione	10,08%	0,43%	4936	6
Sulfamerazine	10,08%	0,41%	5252	204
Moxifloxacin	10,08%	0,43%	4734	209
Ethamsylate	10,08%	0,42%	4932	141
Novobiocin sodium salt	10,07%	0,42%	4886	308
Drofenine hydrochloride	10,07%	0,43%	4231	997
Pepstatin A	10,07%	0,43%	4977	NA
Potassium clavulanate	10,06%	0,41%	4704	837
Tramadol hydrochloride	10,06%	0,41%	5242	134
Closantel	10,06%	0,41%	5347	NA
Mesna	10,06%	0,40%	5134	556
Zimelidine dihydrochloride monohydrate	10,06%	0,43%	4612	368
Dipivefrin hydrochloride	10,05%	0,44%	4476	392
Oxantel pamoate	10,05%	0,39%	5118	1146
Bucladesine sodium salt	10,05%	0,42%	4942	227
Epitiostanol	10,05%	0,41%	5076	250
Mebhydroline 1,5-naphtalenedisulfonate	10,05%	0,41%	5313	NA
Pilocarpine nitrate	10,05%	0,41%	5373	NA
Artemisinin	10,05%	0,49%	3686	78
Nadifloxacin	10,05%	0,45%	4080	409
Lisinopril	10,05%	0,42%	4864	511
Nizatidine	10,05%	0,41%	5143	427
Thyroxine (L)	10,04%	0,52%	3335	NA
Sparfloxacin	10,04%	0,42%	5029	294
Lynestrenol	10,04%	0,44%	4366	401
Balsalazide Sodium	10,04%	0,41%	4195	1503
Flufenamic acid	10,04%	0,40%	5398	310
Exemestane	10,04%	0,42%	4906	261
Indoprofen	10,04%	0,43%	4875	107
Biotin	10,04%	0,44%	4722	NA
Tenoxicam	10,04%	0,42%	5125	79
Denatonium benzoate	10,04%	0,43%	4796	11
Propylthiouracil	10,03%	0,42%	5046	192
Bendroflumethiazide	10,03%	0,44%	3800	1264
Phensuximide	10,03%	0,43%	4768	65
Amiprilose hydrochloride	10,03%	0,41%	5083	426
Sulfabenzamide	10,03%	0,42%	5018	166
Benzthiazide	10,03%	0,42%	5049	249
Moxalactam disodium salt	10,03%	0,42%	5018	54
Nilvadipine	10,02%	0,45%	4449	57
Ethosuximide	10,02%	0,41%	4722	828
Iohexol	10,02%	0,42%	5020	83
Pheniramine maleate	10,02%	0,39%	5742	93
Azelastine HCl	10,02%	0,42%	4966	132
Tranexamic acid	10,02%	0,41%	4817	754
Phenelzine sulfate	10,01%	0,42%	4904	142
Sulfanilamide	10,01%	0,42%	4204	1259
Etofylline	10,01%	0,41%	4961	700
Guanabenz acetate	10,00%	0,46%	4164	161
Procarbazine hydrochloride	10,00%	0,43%	4561	307
Flurandrenolide	10,00%	0,46%	4039	269
Cilostazol	10,00%	0,42%	4896	374
Oxfendazol	9,99%	0,49%	2876	1257
Ifosfamide	9,99%	0,42%	5018	265
Loperamide hydrochloride	9,99%	0,42%	4998	67
Nortriptyline hydrochloride	9,99%	0,43%	4182	897
Catharanthine	9,99%	0,42%	5012	92
Gentamicine sulfate	9,98%	0,39%	5674	460
Tenatoprazole	9,98%	0,42%	5000	251
Acetylcysteine	9,98%	0,39%	5418	586
Dexamethasone acetate	9,97%	0,41%	5326	141
Methapyrilene hydrochloride	9,97%	0,41%	4807	905

Nialamide	9,97%	0,42%	4972	118
Ioxaglic acid	9,97%	0,42%	4992	305
Rufloxacin	9,97%	0,44%	4332	315
Triprolidine hydrochloride	9,96%	0,41%	4940	636
Efavirenz	9,96%	0,43%	4898	NA
Sisomicin sulfate	9,96%	0,42%	5012	28
Rabeprazole Sodium salt	9,95%	0,42%	4820	261
Cefepime hydrochloride	9,95%	0,46%	4080	257
3-alpha-Hydroxy-5-beta-androstan-17-o	9,95%	0,41%	5298	70
Buspirone hydrochloride	9,95%	0,42%	4964	202
Fusidic acid sodium salt	9,94%	0,39%	5420	728
(+,-)-Synephrine	9,94%	0,41%	4994	305
Hydralazine hydrochloride	9,94%	0,43%	4638	298
Chlorpropamide	9,94%	0,42%	4285	1025
Lansoprazole	9,94%	0,42%	4892	139
Glibenclamide	9,94%	0,41%	5354	139
Secnidazole	9,94%	0,41%	5201	16
Deptropine citrate	9,93%	0,43%	4808	63
Acetylsalicylsalicylic acid	9,93%	0,42%	4673	628
Bifonazole	9,93%	0,42%	4819	280
Cefotaxime sodium salt	9,92%	0,41%	5314	95
Suxibuzone	9,92%	0,42%	4958	15
Cephalothin sodium salt	9,92%	0,42%	5078	49
Alfacalcidol	9,92%	0,45%	4184	187
Warfarin	9,91%	0,41%	5116	298
Piracetam	9,91%	0,41%	5158	209
Dydrogesterone	9,91%	0,41%	5154	231
S-(+)-ibuprofen	9,91%	0,41%	5050	252
Mefloquine hydrochloride	9,91%	0,55%	2936	74
Thiocolchicoside	9,90%	0,40%	5400	102
Flumequine	9,90%	0,40%	5502	187
Rivastigmine	9,90%	0,40%	4926	805
Fomepizole	9,90%	0,41%	5175	253
Viomycin sulfate	9,90%	0,43%	4596	186
Dyclonine hydrochloride	9,90%	0,41%	5111	416
Cyclizine hydrochloride	9,90%	0,41%	5220	192
S(-)Eticlopride hydrochloride	9,89%	0,44%	4614	6
Trolox	9,89%	0,40%	5342	250
Riluzole hydrochloride	9,89%	0,41%	4918	564
Yohimbine hydrochloride	9,89%	0,41%	4682	971
Dibucaine	9,89%	0,39%	5606	309
Isoetharine mesylate salt	9,89%	0,43%	3820	1321
Gallamine triethiodide	9,89%	0,42%	4884	174
Thiamine hydrochloride	9,88%	0,41%	5120	186
Bufexamac	9,88%	0,42%	4246	1008
Benzonatate	9,88%	0,40%	5314	185
Griseofulvin	9,88%	0,40%	5112	672
Chlorothiazide	9,88%	0,41%	4736	846
Progesterone	9,88%	0,42%	4980	NA
Suprofen	9,88%	0,42%	5004	81
Penbutolol sulfate	9,87%	0,42%	5008	180
Desipramine hydrochloride	9,87%	0,42%	4678	621
Nefopam hydrochloride	9,87%	0,40%	5609	49
Clofilium tosylate	9,87%	0,42%	4942	292
Felodipine	9,86%	0,42%	5029	NA
Dinoprost trometamol	9,86%	0,41%	5384	NA
Methylprednisolone, 6-alpha	9,86%	0,41%	5242	173
Pergolide mesylate	9,86%	0,41%	5322	60
Maprotiline hydrochloride	9,86%	0,41%	4654	762
Fluconazole	9,86%	0,40%	5316	214
Nylidrin	9,86%	0,44%	4562	18
Oxandrolone	9,86%	0,41%	5153	173
Diphenidol hydrochloride	9,86%	0,43%	4594	276
Cefoperazone dihydrate	9,85%	0,42%	4424	819
Azithromycin	9,85%	0,41%	5072	289
Hydrochlorothiazide	9,85%	0,40%	5450	100
Aceclidine Hydrochloride	9,85%	0,41%	4424	1250
Etodolac	9,85%	0,42%	5058	110
Methylhydantoin-5-(L)	9,85%	0,42%	4985	115
Pimethixene maleate	9,85%	0,40%	5421	303
Granisetron	9,85%	0,39%	5392	633
Penciclovir	9,85%	0,40%	4690	1066
N-Acetyl-DL-homocysteine Thiolactone	9,84%	0,44%	4344	392
Tetracycline hydrochloride	9,84%	0,41%	5246	39
Danazol	9,84%	0,42%	4870	196
Naloxone hydrochloride	9,84%	0,42%	4917	160
Naproxen	9,84%	0,40%	5502	109
Estropipate	9,84%	0,40%	5160	364
Iopanoic acid	9,84%	0,42%	4975	51
Amifostine	9,84%	0,46%	4158	NA
6-Furfurylaminopurine	9,84%	0,42%	5003	171
Ropinirole HCl	9,83%	0,42%	4906	53
Tolazamide	9,83%	0,41%	4992	265
Theophylline monohydrate	9,83%	0,42%	4820	293
Diethylcarbamazine citrate	9,83%	0,40%	5460	78
Pyrithyldione	9,82%	0,41%	5124	53
Halcinonide	9,82%	0,42%	4864	104
Clocortolone pivalate	9,82%	0,44%	4440	57
Chloramphenicol	9,82%	0,41%	5046	309
Zaleplon	9,82%	0,42%	5046	108
Proadifen hydrochloride	9,81%	0,42%	4112	1246
Scopolamine hydrochloride	9,81%	0,42%	4900	122
Sulfacetamide sodic hydrate	9,81%	0,40%	5592	38
Indomethacin	9,80%	0,40%	5322	435
Adrenosterone	9,80%	0,42%	4944	4
Cinoxacin	9,80%	0,42%	4091	1341
Acamprosate calcium	9,80%	0,40%	5432	267
Alcuronium chloride	9,80%	0,42%	5074	9
Gliquidone	9,80%	0,42%	4874	63
Oxaprozin	9,80%	0,41%	5101	177
Cefmetazole sodium salt	9,80%	0,41%	5078	138
Carbenoxolone disodium salt	9,80%	0,43%	4772	108
Doxepin hydrochloride	9,79%	0,42%	5000	105
Clomiphene citrate (Z,E)	9,79%	0,46%	3934	242
Fursultiamine Hydrochloride	9,79%	0,40%	5294	298
Diflunisal	9,79%	0,39%	5601	112
Dichlorphenamide	9,79%	0,41%	5166	64
Carbadox	9,79%	0,42%	5056	74
Metoclopramide monohydrochloride	9,79%	0,40%	5473	78
Amlodipine	9,79%	0,46%	4251	NA
Rifapentine	9,78%	0,42%	5100	NA
Benfotiamine	9,78%	0,41%	5239	81
Gabapentin	9,78%	0,43%	4683	218
Opipramol dihydrochloride	9,78%	0,41%	5274	49
Bromperidol	9,78%	0,43%	4696	228
Prothionamide	9,78%	0,39%	5362	431
Hydroflumethiazide	9,77%	0,40%	5369	91
(+,-)-Octopamine hydrochloride	9,77%	0,42%	4900	90
Chlorphensin carbamate	9,77%	0,40%	5340	237
Tetrahydrozoline hydrochloride	9,77%	0,41%	4951	238

Ethopropazine hydrochloride	9,77%	0,41%	4980	196
Allantoin	9,77%	0,40%	5554	124
Clofibric acid	9,76%	0,44%	3890	972
Cloxacillin sodium salt	9,76%	0,41%	5240	21
Norgestrel-(-)-D	9,76%	0,43%	3913	1056
Sulfadimethoxine	9,76%	0,43%	4050	1169
Dorzolamide hydrochloride	9,76%	0,44%	4051	631
Bemegride	9,76%	0,40%	5216	274
Famprofazone	9,76%	0,43%	4665	40
Clemizole hydrochloride	9,75%	0,41%	5158	26
Dipyridamole	9,75%	0,41%	5048	445
Guanethidine sulfate	9,75%	0,42%	4872	288
Mefenamic acid	9,75%	0,40%	5348	170
Dosulepin hydrochloride	9,75%	0,47%	3815	221
Flecainide acetate	9,74%	0,43%	4084	1075
Dihydrostreptomycin sulfate	9,74%	0,40%	5248	344
Cinnarizine	9,74%	0,44%	4378	272
Quetiapine	9,73%	0,44%	4592	NA
Oxymetazoline hydrochloride	9,73%	0,40%	5432	194
Flunixin meglumine	9,73%	0,43%	4024	996
Cyproheptadine hydrochloride	9,72%	0,41%	5010	204
Corticosterone	9,72%	0,43%	4794	NA
Tetraethylenepentamine pentahydrochlo	9,72%	0,42%	4756	170
Guaiacol	9,72%	0,40%	5439	37
Norfloxacin	9,71%	0,43%	4756	44
Formoterol fumarate	9,71%	0,42%	4412	749
Loxapine succinate	9,71%	0,41%	5082	240
Adenosine 5'-monophosphate monohyd	9,70%	0,41%	4950	347
Methimazole	9,70%	0,42%	4042	1398
Alendronate sodium	9,70%	0,46%	3134	1531
Zoxazolamine	9,70%	0,42%	4156	1138
Oxacillin sodium	9,69%	0,40%	5403	47
Pyrimethamine	9,69%	0,48%	3061	938
Dizocilpine maleate	9,69%	0,41%	5088	172
Ambrisentan	9,69%	0,42%	4796	222
Sulfaquinoxaline sodium salt	9,69%	0,43%	4069	1078
Fenoprofen calcium salt dihydrate	9,69%	0,42%	4172	1107
Proglumide	9,69%	0,39%	5554	278
Azathioprine	9,68%	0,53%	2896	387
Ethoxyquin	9,68%	0,44%	3820	995
Tiapride hydrochloride	9,68%	0,39%	5574	136
Zaprinast	9,68%	0,40%	4650	1137
Clotrimazole	9,68%	0,45%	4138	385
Camylofine chlorhydrate	9,68%	0,41%	5148	53
Adiphenine hydrochloride	9,68%	0,40%	5509	55
Meptazinol hydrochloride	9,68%	0,41%	5164	76
Acemetacin	9,68%	0,40%	5346	125
Apramycin	9,67%	0,41%	5166	78
Anastrozole	9,67%	0,39%	5489	529
Isopropamide iodide	9,67%	0,41%	4232	1312
Moroxidine hydrochloride	9,67%	0,47%	3785	132
Hyoscyamine (L)	9,67%	0,40%	5352	52
Nadide	9,67%	0,40%	4408	1607
Tacrine hydrochloride	9,66%	0,42%	4456	693
Phenethicillin potassium salt	9,66%	0,40%	4358	1534
(R) -Naproxen sodium salt	9,66%	0,42%	4036	1377
Cefotetan	9,66%	0,44%	4410	NA
Ethisterone	9,66%	0,41%	4652	844
Arbutin	9,66%	0,42%	4920	71
Epirizole	9,66%	0,40%	5472	67
Azapropazone	9,66%	0,41%	5206	122
Leflunomide	9,66%	0,41%	4345	1047
Nalidixic acid sodium salt	9,66%	0,40%	5282	74
Glimepiride	9,65%	0,41%	5116	16
Haloperidol	9,65%	0,41%	4931	191
Selegiline hydrochloride	9,65%	0,42%	4562	655
(S)-(-)-Atenolol	9,65%	0,41%	5204	11
Triamterene	9,65%	0,41%	4981	431
Buflomedil hydrochloride	9,65%	0,42%	4944	NA
Hydroxytacrine maleate (R,S)	9,64%	0,41%	5258	NA
Ampicillin trihydrate	9,64%	0,41%	5064	128
Chlorcyclizine hydrochloride	9,64%	0,41%	5164	179
Androsterone	9,64%	0,45%	4389	NA
(+) -Levobunolol hydrochloride	9,64%	0,41%	4935	197
Darifenacin hydrobromide	9,64%	0,42%	5034	13
Carteolol hydrochloride	9,63%	0,42%	4994	NA
Metyrapone	9,63%	0,40%	5392	85
Benzylpenicillin sodium	9,63%	0,40%	5132	322
Alverine citrate salt	9,63%	0,40%	4594	1009
Imiquimod	9,62%	0,41%	4747	486
Ribostamycin sulfate salt	9,62%	0,42%	4210	1145
Mafenide hydrochloride	9,62%	0,41%	4956	284
Tranilast	9,62%	0,42%	4930	29
Methenamine	9,62%	0,42%	4949	31
Amidopyrine	9,61%	0,44%	3997	673
Primidone	9,61%	0,41%	4885	477
Mephenytoin	9,61%	0,43%	4764	60
Methotrimeprazine maleat salt	9,61%	0,45%	3614	1056
Tropisetron HCl	9,61%	0,44%	4433	NA
Vigabatrin	9,61%	0,43%	4650	180
Nadolol	9,61%	0,41%	4912	235
Sulconazole nitrate	9,61%	0,43%	4582	72
Quinethazone	9,60%	0,42%	4898	185
Glutethimide, para-amino	9,60%	0,40%	4526	1199
Fluocinolone acetonide	9,60%	0,39%	4976	856
Tremorine dihydrochloride	9,60%	0,39%	4838	1067
Tridihexethyl chloride	9,60%	0,41%	5000	59
Doxazosin mesylate	9,60%	0,42%	4752	248
Clopamide	9,60%	0,39%	5238	842
Methocarbamol	9,60%	0,40%	5203	290
Triamcinolone	9,59%	0,40%	5287	232
Mepenzolate bromide	9,59%	0,42%	4986	54
Fulvestrant	9,59%	0,46%	3750	521
Bephenium hydroxynaphthoate	9,59%	0,41%	5007	59
Diloxanide furoate	9,58%	0,42%	4926	88
Canrenone	9,58%	0,39%	5205	608
Pempidine tartrate	9,58%	0,40%	5235	345
Sulfamethizole	9,58%	0,41%	4339	1259
Flunisolide	9,58%	0,45%	4276	79
Levetiracetam	9,58%	0,42%	4583	486
Ritodrine hydrochloride	9,58%	0,41%	4682	598
Aminohippuric acid	9,58%	0,39%	5360	333
Torsemide	9,58%	0,41%	4900	276
Debrisoquin sulfate	9,58%	0,43%	3990	1091
Carprofen	9,57%	0,39%	5289	526
Miconazole	9,57%	0,42%	4488	728
Roxarsone	9,57%	0,40%	5246	204
Amoxapine	9,57%	0,43%	4652	144
Minocycline hydrochloride	9,57%	0,49%	2924	920
Valproic acid	9,57%	0,40%	4822	659

Methacholine chloride	9,57%	0,43%	4015	1032
Cisapride	9,57%	0,41%	5174	NA
Clindamycin hydrochloride	9,56%	0,40%	5283	235
Pyridostigmine iodid	9,56%	0,39%	5431	263
Fluorometholone	9,56%	0,40%	4795	737
Tocainide hydrochloride	9,55%	0,42%	4782	17
Picrotoxinin	9,55%	0,41%	5068	12
Meticrane	9,55%	0,38%	5673	445
Fosfosal	9,55%	0,41%	4936	350
Midodrine hydrochloride	9,55%	0,41%	5078	15
Phentermine hydrochloride	9,55%	0,41%	4916	305
Isoquinoline, 6,7-dimethoxy-1-methyl-1,2	9,55%	0,41%	4942	165
Acenocoumarol	9,54%	0,41%	5062	193
Zafirlukast	9,54%	0,41%	4930	217
Furosemide	9,54%	0,41%	4605	735
Hexylcaine hydrochloride	9,54%	0,43%	3920	1143
Gabexate mesilate	9,54%	0,40%	5218	152
Tolbutamide	9,54%	0,40%	5284	326
Nimesulide	9,54%	0,39%	5340	276
Ketoprofen	9,54%	0,39%	5697	144
Mianserine hydrochloride	9,53%	0,42%	4868	161
Nafronyl oxalate	9,53%	0,39%	5134	557
4-aminosalicylic acid	9,53%	0,43%	4465	139
Amiloride hydrochloride dihydrate	9,53%	0,40%	5300	56
Valacyclovir hydrochloride	9,53%	0,40%	5320	NA
Thalidomide	9,53%	0,41%	4874	378
Fosinopril	9,53%	0,42%	4837	47
Finasteride	9,53%	0,40%	5256	20
Roxatidine Acetate HCl	9,52%	0,41%	5134	NA
Fluvoxamine maleate	9,52%	0,40%	5040	519
Emedastine	9,52%	0,42%	4876	23
Vardenafil	9,52%	0,39%	5430	190
Cyclobenzaprine hydrochloride	9,52%	0,41%	5179	NA
Sulfinpyrazone	9,52%	0,40%	4942	573
Tobramycin	9,51%	0,42%	4752	176
Famotidine	9,51%	0,41%	5043	153
Budesonide	9,51%	0,45%	2863	2077
Citalopram Hydrobromide	9,51%	0,39%	5556	83
Alprostadil	9,51%	0,40%	5168	188
THIP Hydrochloride	9,51%	0,40%	4971	617
Busulfan	9,51%	0,42%	4677	259
Morantel tartrate	9,50%	0,40%	4946	492
Sulfadoxine	9,50%	0,43%	4349	369
Salbutamol	9,50%	0,42%	4694	210
Mometasone furoate	9,50%	0,43%	3952	1006
Mexiletine hydrochloride	9,49%	0,41%	4118	1357
Biperiden hydrochloride	9,49%	0,41%	4930	105
Phenylbutazone	9,49%	0,39%	5632	38
Felbinac	9,49%	0,41%	4870	344
Gestrinone	9,48%	0,41%	5072	148
Pranlukast	9,48%	0,39%	5633	74
(S)-propranolol hydrochloride	9,48%	0,40%	5054	535
Spaglumic acid	9,48%	0,40%	5130	174
Triflusal	9,48%	0,39%	5364	276
Rolipram	9,48%	0,42%	4717	267
Folic acid	9,47%	0,41%	4694	511
Ethambutol dihydrochloride	9,47%	0,39%	5530	107
Benzydamine hydrochloride	9,47%	0,40%	4494	1083
Atropine sulfate monohydrate	9,47%	0,41%	4326	1187
Cyclopenthiazide	9,46%	0,39%	5464	269
Isometheptene mucate	9,46%	0,41%	5119	99
Formestane	9,46%	0,42%	4800	75
Methyldopate hydrochloride	9,45%	0,41%	4958	124
Piperacetazine	9,45%	0,43%	4664	32
Thioperamide maleate	9,45%	0,40%	4920	569
Hexamethonium dibromide dihydrate	9,45%	0,39%	5559	129
Prednisone	9,45%	0,39%	5427	301
Estriol	9,45%	0,41%	4766	310
Sulmazole	9,45%	0,45%	4104	288
Quipazine dimaleate salt	9,45%	0,40%	5312	153
Vincamine	9,45%	0,40%	4883	520
Naftifine hydrochloride	9,44%	0,42%	4778	146
Fluticasone propionate	9,44%	0,39%	5302	416
Thiorphan	9,43%	0,42%	4348	733
Pancuronium bromide	9,43%	0,42%	4601	195
Propafenone hydrochloride	9,43%	0,44%	4450	4
Todralazine hydrochloride	9,43%	0,39%	5468	37
Sulfamethoxazole	9,43%	0,42%	2694	3181
Equilin	9,42%	0,40%	5222	16
Omeprazole	9,42%	0,42%	4710	305
Diethylstilbestrol	9,42%	0,44%	3842	814
Flucloxacillin sodium	9,42%	0,39%	5524	177
Alfuzosin hydrochloride	9,41%	0,40%	4370	1240
Montelukast	9,41%	0,41%	4838	262
Phenindione	9,41%	0,40%	5283	117
Imipramine hydrochloride	9,41%	0,40%	5236	214
Amitryptiline hydrochloride	9,41%	0,40%	5296	190
Benfluorex hydrochloride	9,41%	0,39%	5276	655
Levopropoxyphene napsylate	9,40%	0,39%	5322	284
Cloperastine hydrochloride	9,39%	0,43%	3718	1170
Bromhexine hydrochloride	9,39%	0,42%	4674	271
Brinzolamide	9,39%	0,39%	5122	832
Amrinone	9,39%	0,40%	4180	1473
Ioversol	9,38%	0,41%	4907	204
Sumatriptan succinate	9,38%	0,39%	5510	196
Clobutinol hydrochloride	9,38%	0,46%	4007	NA
Temozolomide	9,38%	0,41%	5082	19
Miglitol	9,38%	0,41%	4908	70
Ranitidine hydrochloride	9,38%	0,41%	4922	51
Ambroxol hydrochloride	9,38%	0,38%	5429	723
Sertraline	9,38%	0,45%	3816	637
Butenafine Hydrochloride	9,38%	0,40%	5191	140
Fluocinonide	9,38%	0,43%	3898	1078
Bisacodyl	9,38%	0,44%	4384	NA
Dantrolene sodium salt	9,37%	0,42%	4607	245
Scopolamin-N-oxide hydrobromide	9,37%	0,40%	5162	396
Saquinavir mesylate	9,37%	0,44%	4207	314
Isoniazid	9,37%	0,40%	4586	1091
Argatroban	9,37%	0,39%	5303	546
Nicergoline	9,37%	0,43%	4297	354
Bromocryptine mesylate	9,37%	0,45%	4100	44
Tulobuterol	9,37%	0,42%	4700	86
Phthalylsulfathiazole	9,37%	0,41%	4914	251
Tibolone	9,37%	0,40%	5218	63
Olmesartan	9,36%	0,43%	4466	178
Pramoxine hydrochloride	9,36%	0,40%	5024	311
Dicloxacillin sodium salt hydrate	9,36%	0,39%	5458	NA
Imipenem	9,36%	0,40%	5190	139
Furazolidone	9,36%	0,42%	4736	224
Estrone	9,36%	0,40%	4972	282

Metaproterenol sulfate, orciprenaline sul	9,36%	0,44%	4340	13
Dobutamine hydrochloride	9,35%	0,42%	4446	394
Clonixin Lysinate	9,35%	0,43%	4484	54
Sulindac	9,35%	0,39%	5450	339
Ciprofloxacin hydrochloride hydrate	9,35%	0,40%	5094	268
Sulfisoxazole	9,35%	0,39%	4830	1052
Althiazide	9,35%	0,44%	3822	861
Lymecycline	9,34%	0,39%	5505	146
Diosmin	9,34%	0,41%	4804	501
Famciclovir	9,34%	0,40%	5205	314
Amiodarone hydrochloride	9,34%	0,47%	3769	NA
Ornidazole	9,33%	0,39%	5213	324
Iodixanol	9,33%	0,40%	5100	229
Eucatropine hydrochloride	9,33%	0,42%	3966	1016
Etofenamate	9,32%	0,42%	4780	37
Ipsapirone	9,32%	0,40%	5250	115
Urosiol	9,32%	0,39%	5028	645
Methoxamine hydrochloride	9,32%	0,40%	5266	202
Domperidone	9,32%	0,40%	4938	349
Mefexamide hydrochloride	9,32%	0,39%	3693	2489
Trimetazidine dihydrochloride	9,31%	0,39%	5390	106
Tetramisole hydrochloride	9,31%	0,39%	5336	153
Amprolium hydrochloride	9,31%	0,39%	5496	169
Panthenol (D)	9,31%	0,39%	5485	262
Isocarboxazid	9,31%	0,43%	3738	1199
Furaltadone hydrochloride	9,31%	0,42%	3948	1039
Sulfamethazine sodium salt	9,31%	0,44%	3787	873
Primaquine diphosphate	9,31%	0,41%	4910	NA
Dimaprit dihydrochloride	9,31%	0,40%	5206	159
Piribedil hydrochloride	9,30%	0,39%	5474	101
Sulfaphenazole	9,30%	0,39%	5468	292
Carvedilol	9,30%	0,43%	4470	261
Cefotiam hydrochloride	9,30%	0,40%	5280	91
Clarithromycin	9,29%	0,39%	5306	453
Succinylsulfathiazole	9,29%	0,50%	3174	274
Xamoterol hemifumarate	9,29%	0,40%	5195	141
Acetazolamide	9,29%	0,40%	5286	60
Imidurea	9,29%	0,40%	5193	225
Celiprolol HCl	9,29%	0,41%	5016	95
Ceforanide	9,29%	0,40%	5223	NA
Sulfamethoxypyridazine	9,29%	0,40%	4176	1374
Lithocholic acid	9,28%	0,42%	4026	964
Celecoxib	9,28%	0,42%	4535	492
Lofexidine	9,28%	0,44%	4413	25
Hemicholinium bromide	9,27%	0,50%	3263	98
Isosorbide dinitrate	9,27%	0,39%	5292	252
Fluphenazine dihydrochloride	9,27%	0,42%	4696	226
Ethinylestradiol	9,26%	0,50%	2964	558
Pioglitazone	9,26%	0,41%	4934	47
Clomipramine hydrochloride	9,25%	0,42%	4678	19
Melatonin	9,25%	0,39%	5407	NA
Stanozolol	9,25%	0,41%	4943	NA
Beta-Escin	9,24%	0,43%	3594	1481
Topiramate	9,24%	0,41%	4869	122
(-) -Levobunolol hydrochloride	9,24%	0,41%	4899	188
Bumetanide	9,24%	0,40%	5188	25
Neostigmine bromide	9,24%	0,39%	5401	NA
Pentolinium bitartrate	9,24%	0,39%	5336	185
Piretanide	9,24%	0,41%	4788	286
Tiabendazole	9,24%	0,41%	4838	293
Nitrocaramiphen hydrochloride	9,23%	0,39%	5588	77
Oxolinic acid	9,23%	0,39%	5330	381
Carbinoxamine maleate salt	9,23%	0,43%	4264	434
Vinpocetine	9,23%	0,40%	5074	71
Cephalosporanic acid, 7-amino	9,23%	0,42%	4821	NA
Diflorasone Diacetate	9,22%	0,38%	5670	62
Pentetic acid	9,22%	0,42%	4766	7
Anthralin	9,22%	0,40%	4999	199
Fipexide hydrochloride	9,21%	0,41%	5034	5
Troglitazone	9,20%	0,44%	4064	474
Caffeine	9,20%	0,41%	4808	145
Rofecoxib	9,20%	0,40%	5167	71
Nitrofurantoin	9,20%	0,41%	4432	665
2-Chloropyrazine	9,20%	0,40%	4952	207
Pentylenetetrazole	9,19%	0,40%	4522	897
Clodronate	9,19%	0,40%	5130	175
Crotamiton	9,19%	0,40%	5144	51
Nitrendipine	9,19%	0,41%	4962	78
Clonidine hydrochloride	9,18%	0,39%	4433	1476
Penicillamine	9,18%	0,39%	5438	256
Reboxetine mesylate	9,18%	0,40%	5028	377
Betahistine mesylate	9,18%	0,40%	5058	382
Irsogladine maleate	9,18%	0,40%	5084	131
Etanidazole	9,17%	0,43%	4332	182
Dexfenfluramine hydrochloride	9,17%	0,38%	4817	1242
(S)-(-)-Cycloserine	9,16%	0,39%	5370	153
Hydroxychloroquine sulfate	9,16%	0,38%	5520	192
Lorglumide sodium salt	9,16%	0,40%	5094	238
Dopamine hydrochloride	9,16%	0,39%	5150	374
Chlormezanone	9,16%	0,40%	4660	853
Nalmefene hydrochloride	9,16%	0,40%	5142	187
Diprophylline	9,16%	0,38%	5419	414
D-cycloserine	9,15%	0,41%	4784	186
Enilconazole	9,15%	0,39%	5250	424
Levamisole hydrochloride	9,15%	0,40%	5002	344
(-)-Eseroline fumarate salt	9,15%	0,41%	4658	368
Flucytosine	9,14%	0,40%	5000	107
Carbidopa	9,14%	0,41%	4900	112
Hesperidin	9,13%	0,39%	5178	497
Alfadolone acetate	9,13%	0,39%	5277	96
Colistin sulfate	9,13%	0,41%	4684	473
Amyleine hydrochloride	9,13%	0,38%	5728	190
Glafenine hydrochloride	9,12%	0,41%	4522	612
Pridinol methanesulfonate salt	9,12%	0,41%	4166	1163
Hymecromone	9,12%	0,40%	5044	29
Sotalol hydrochloride	9,12%	0,40%	5042	111
Meprylcaine hydrochloride	9,12%	0,39%	5134	308
Idazoxan hydrochloride	9,11%	0,40%	4972	404
Amikacin hydrate	9,11%	0,39%	4814	1030
Pyridoxine hydrochloride	9,11%	0,40%	5046	223
Terbutaline hemisulfate	9,11%	0,48%	3178	582
Benazepril HCl	9,10%	0,40%	5124	147
Streptomycin sulfate	9,10%	0,41%	4072	1296
Ibutilide fumarate	9,10%	0,40%	5086	16
Esmolol hydrochloride	9,10%	0,40%	4936	214
Trimethoprim	9,10%	0,38%	5474	310
Pipenzolate bromide	9,10%	0,40%	4312	1240
Clemastine fumarate	9,09%	0,39%	5172	325
Cefuroxime sodium salt	9,09%	0,39%	5134	507
Theobromine	9,08%	0,41%	4934	107

Diphenylpyraline hydrochloride	9,08%	0,39%	5138	244
Captopril	9,07%	0,40%	5190	17
Propoxycaine hydrochloride	9,07%	0,40%	5048	312
Dilazep dihydrochloride	9,07%	0,39%	5380	1
Tazobactam	9,07%	0,40%	5192	2
Piroxicam	9,07%	0,40%	4900	383
Meloxicam	9,07%	0,39%	5074	622
Ranolazine	9,07%	0,39%	5260	299
Promazine hydrochloride	9,06%	0,42%	4594	42
Levalbuterol hydrochloride	9,06%	0,42%	4591	188
Loracarbef	9,06%	0,39%	5190	139
Phenacetin	9,06%	0,41%	4820	54
Bromopride	9,05%	0,41%	4944	12
Dextromethorphan hydrobromide monol	9,04%	0,38%	5224	872
Niridazole	9,03%	0,39%	5480	NA
Diperodon hydrochloride	9,03%	0,40%	5116	14
Verapamil hydrochloride	9,03%	0,40%	4436	914
Molsidomine	9,03%	0,39%	5025	412
Memantine Hydrochloride	9,02%	0,39%	5342	171
Fleroxacin	9,02%	0,40%	4447	810
Tetracaïne hydrochloride	9,02%	0,40%	5032	77
Mesalamine	9,02%	0,39%	5122	235
Nandrolone	9,02%	0,39%	5420	158
Carbachol	9,02%	0,40%	4990	203
Levocabastine hydrochloride	9,02%	0,40%	4978	190
Calcipotriene	9,01%	0,45%	4017	NA
Tribenoside	9,01%	0,40%	4788	371
GBR 12909 dihydrochloride	9,01%	0,41%	4558	338
Altretamine	9,00%	0,40%	4734	467
Deoxycorticosterone	9,00%	0,41%	4668	405
2-Aminobenzenesulfonamide	9,00%	0,40%	5028	230
Vatalanib	9,00%	0,46%	3935	NA
Zalcitabine	8,99%	0,42%	4150	549
Toltrazuril	8,99%	0,41%	4850	172
Ofloxacin	8,99%	0,39%	4928	566
Doxofylline	8,99%	0,39%	5272	113
Tolfenamic acid	8,99%	0,38%	5286	455
Flurbiprofen	8,99%	0,41%	4788	43
Isradipine	8,98%	0,40%	5114	8
Pivampicillin	8,98%	0,37%	5470	530
Econazole nitrate	8,98%	0,40%	4802	278
Diclazuril	8,98%	0,40%	4922	329
Xylometazoline hydrochloride	8,98%	0,37%	5838	231
Lomefloxacin hydrochloride	8,98%	0,41%	4774	272
Antipyrine	8,98%	0,38%	5424	141
Tiratricol, 3,3',5-triiodothyroacetic acid	8,98%	0,39%	5290	50
Vecuronium bromide	8,97%	0,41%	4615	344
Benztropine mesylate	8,97%	0,40%	4806	487
Glycopyrrolate	8,97%	0,40%	4260	1277
Halofantrine hydrochloride	8,97%	0,44%	4130	152
Fenoterol hydrobromide	8,97%	0,41%	4720	138
Clorgyline hydrochloride	8,96%	0,39%	4671	888
Nisoxetine hydrochloride	8,96%	0,40%	5072	135
Doxycycline hydrochloride	8,96%	0,41%	4740	151
Butylparaben	8,96%	0,41%	4834	95
Anethole-trithione	8,95%	0,40%	4960	62
Diphemanil methylsulfate	8,95%	0,37%	5696	243
Pirlindole mesylate	8,94%	0,38%	5298	571
Fenbufen	8,94%	0,38%	5594	42
Trihexyphenidyl-D,L Hydrochloride	8,94%	0,40%	4816	264
Olopatadine hydrochloride	8,93%	0,39%	5089	260
Nefazodone HCl	8,93%	0,38%	5047	658
Serotonin hydrochloride	8,93%	0,40%	4528	743
Meglumine	8,92%	0,40%	5090	138
Cyclopentolate hydrochloride	8,92%	0,43%	4432	41
Tomoxetine hydrochloride	8,92%	0,39%	4552	999
Minaprine dihydrochloride	8,92%	0,38%	5629	65
Iopamidol	8,92%	0,40%	5076	180
Meclofenamic acid sodium salt monohyc	8,91%	0,38%	5472	325
Toremifene	8,91%	0,42%	4436	139
Chloroquine diphosphate	8,91%	0,38%	5292	311
Oxytetracycline dihydrate	8,90%	0,39%	5046	240
Testosterone propionate	8,90%	0,44%	4137	NA
Pravastatin	8,89%	0,41%	4284	875
(-)-MK 801 hydrogen maleate	8,89%	0,39%	4718	887
Droperidol	8,88%	0,38%	5303	334
Ibudilast	8,87%	0,42%	4402	264
Sarafloxacin	8,87%	0,40%	4804	223
Hexestrol	8,86%	0,40%	4930	36
Sulfachloropyridazine	8,86%	0,41%	4504	319
Sulfamonomethoxine	8,85%	0,40%	4930	201
Piperidolate hydrochloride	8,85%	0,39%	5006	319
Monobenzone	8,85%	0,45%	3338	904
Loratadine	8,85%	0,39%	4970	386
Pyrazinamide	8,84%	0,40%	4863	222
Oxiconazole Nitrate	8,84%	0,44%	3804	443
Amodiaquin dihydrochloride dihydrate	8,84%	0,38%	5345	363
Ticarcillin sodium	8,83%	0,39%	5122	87
Acipimox	8,83%	0,38%	5399	161
Luteolin	8,83%	0,50%	3089	178
Zileuton	8,83%	0,39%	5189	139
Decamethonium bromide	8,83%	0,39%	5298	35
Galanthamine hydrobromide	8,83%	0,39%	5195	55
Cefaclor hydrate	8,83%	0,39%	5324	17
Desloratadine	8,82%	0,40%	4776	463
Methiothepin maleate	8,81%	0,41%	4509	223
Etomidate	8,81%	0,40%	4936	150
Perhexiline maleate	8,80%	0,40%	4142	1145
Atracurium besylate	8,80%	0,40%	4936	151
Pizotifen malate	8,80%	0,39%	5276	160
Pimozide	8,79%	0,45%	3300	899
Repaglinide	8,79%	0,41%	4684	262
Sulfameter	8,79%	0,41%	4016	1063
Moricizine hydrochloride	8,79%	0,41%	4790	84
Mirtazapine	8,78%	0,38%	5072	783
Butylscopolammonium (n-) bromide	8,78%	0,39%	5026	252
Butalbital	8,78%	0,38%	5480	NA
Urapidil hydrochloride	8,76%	0,39%	5096	56
Mebeverine hydrochloride	8,76%	0,39%	5236	5
Atovaquone	8,76%	0,39%	4996	265
Chicago sky blue 6B	8,76%	0,38%	5596	NA
Mizolastine	8,76%	0,38%	5120	497
Zotepine	8,75%	0,47%	3633	NA
Metronidazole	8,75%	0,43%	3662	880
Amcinonide	8,75%	0,40%	5108	0
Clofibrate	8,75%	0,40%	4815	208
Pirenperone	8,74%	0,40%	4988	78
Promethazine hydrochloride	8,74%	0,40%	4916	18
Ganciclovir	8,74%	0,41%	4714	128
Josamycin	8,74%	0,41%	4701	99

Bosentan	8,74%	0,46%	3382	477
Talampicillin hydrochloride	8,74%	0,38%	5608	64
Gliclazide	8,73%	0,38%	5314	233
Iodipamide	8,73%	0,39%	5006	166
Zidovudine, AZT	8,72%	0,39%	4485	984
Erlotinib	8,72%	0,49%	3279	NA
L(-)-vesamicol hydrochloride	8,72%	0,37%	5448	421
Sulbactam	8,72%	0,39%	4941	296
Etidronic acid, disodium salt	8,72%	0,39%	5014	307
Ondansetron Hydrochloride	8,70%	0,39%	5077	86
Benzbromarone	8,70%	0,38%	5206	622
Nilutamide	8,70%	0,40%	4912	47
Chlorprothixene hydrochloride	8,69%	0,41%	4474	326
Cyclosporin A	8,69%	0,49%	3371	NA
Norcyclobenzaprine	8,69%	0,41%	4558	145
Milrinone	8,69%	0,39%	5082	171
Mepivacaine hydrochloride	8,69%	0,39%	4686	845
Dirithromycin	8,69%	0,41%	4814	15
Homosalate	8,68%	0,40%	4892	216
Allopurinol	8,68%	0,40%	4794	114
Melengestrol acetate	8,66%	0,41%	4551	143
N-Acetyl-L-leucine	8,66%	0,39%	5158	86
Sulfadiazine	8,66%	0,37%	5592	212
Niacin	8,65%	0,41%	4640	139
Dimethisoquin hydrochloride	8,65%	0,40%	5048	6
Meclocycline sulfosalicylate	8,65%	0,44%	4071	NA
Methacycline hydrochloride	8,64%	0,40%	4955	76
Acetylsalicylic acid	8,64%	0,38%	5186	375
Diacerein	8,64%	0,38%	5284	207
Aminopurine, 6-benzyl	8,64%	0,40%	4992	80
Oxyphenbutazone	8,63%	0,39%	4944	185
Brompheniramine maleate	8,63%	0,43%	4241	NA
Guanfacine hydrochloride	8,63%	0,40%	4491	453
Stavudine	8,62%	0,39%	4770	409
Bisoprolol fumarate	8,61%	0,39%	4618	939
Clorsulon	8,61%	0,37%	5640	4
Mecamylamine hydrochloride	8,60%	0,39%	5011	133
Prednicarbate	8,60%	0,38%	5133	307
Zuclopenthixol hydrochloride	8,59%	0,39%	4700	627
Trifluoperazine dihydrochloride	8,59%	0,50%	2634	639
Mifepristone	8,59%	0,41%	4544	177
Isoconazole	8,58%	0,39%	4937	267
Aminocaproic acid	8,58%	0,40%	4644	223
Methylatropine nitrate	8,57%	0,39%	5081	107
Cefoxitin sodium salt	8,56%	0,39%	5024	196
Chlormadinone acetate	8,56%	0,39%	5085	146
Paroxetine Hydrochloride	8,56%	0,40%	4844	57
Clinafloxacin	8,55%	0,39%	5006	297
Fluoxetine hydrochloride	8,55%	0,43%	4111	44
Lamotrigine	8,54%	0,37%	5442	419
Risperidone	8,53%	0,40%	4940	45
Prazosin hydrochloride	8,53%	0,42%	3544	1357
(+)-Isoproterenol (+)-bitartrate salt	8,52%	0,41%	4520	179
Trioxsalen	8,52%	0,39%	4974	154
Clebopride maleate	8,51%	0,37%	5206	648
Rifabutin	8,51%	0,42%	4473	57
Fentiazac	8,50%	0,44%	3975	NA
Pregnenolone	8,50%	0,38%	5150	187
Mesoridazine besylate	8,49%	0,38%	5250	124
Nimodipine	8,49%	0,39%	5126	32
Nabumetone	8,48%	0,38%	5162	251
Phenazopyridine hydrochloride	8,48%	0,39%	4250	1231
(R)-(+)-Atenolol	8,47%	0,40%	4791	66
Abacavir Sulfate	8,47%	0,40%	4927	8
Butamben	8,47%	0,40%	4010	1357
Nitrofural	8,47%	0,40%	4672	355
Aniracetam	8,47%	0,38%	5364	80
Ceftazidime pentahydrate	8,46%	0,41%	4618	86
Tiletamine hydrochloride	8,45%	0,45%	3472	597
Pralidoxime chloride	8,44%	0,40%	4869	55
Quinapril HCl	8,43%	0,38%	4988	361
DO 897/99	8,42%	0,38%	5196	128
Prenylamine lactate	8,41%	0,41%	4570	79
Clozapine	8,40%	0,38%	5122	397
(R)-Propranolol hydrochloride	8,40%	0,40%	4815	7
Sildenafil	8,40%	0,39%	4591	711
Dioxybenzone	8,40%	0,38%	5142	271
Nomegestrol acetate	8,40%	0,40%	4538	269
Cyclophosphamide	8,38%	0,37%	5194	682
(-)-Isoproterenol hydrochloride	8,37%	0,41%	4603	31
Fendiline hydrochloride	8,35%	0,40%	4742	4
Rebamipide	8,34%	0,41%	4366	384
Losartan	8,32%	0,39%	4544	647
Clenbuterol hydrochloride	8,31%	0,40%	4340	617
Thioridazine hydrochloride	8,30%	0,40%	3963	1247
Ibandronate sodium	8,29%	0,38%	5110	107
Remoxipride Hydrochloride	8,29%	0,38%	5284	15
Oxybenzone	8,29%	0,38%	4828	523
Proparacaine hydrochloride	8,29%	0,39%	4842	198
Thiethylperazine dimalate	8,28%	0,39%	4872	78
Cetirizine dihydrochloride	8,27%	0,39%	4950	146
Etoricoxib	8,27%	0,38%	5077	242
Estradiol Valerate	8,27%	0,39%	4744	320
Benserazide hydrochloride	8,26%	0,38%	5090	113
Propofol	8,24%	0,38%	5208	81
Dolasetron mesilate	8,24%	0,39%	4900	37
Triflupromazine hydrochloride	8,24%	0,39%	4580	492
Bepridil hydrochloride	8,23%	0,39%	4504	603
Telmisartan	8,21%	0,40%	4730	132
Rifaximin	8,19%	0,39%	4720	405
Ivermectin	8,18%	0,40%	3835	1386
Cilnidipine	8,17%	0,40%	4752	28
Terazosin hydrochloride	8,16%	0,39%	4104	1195
Ketorolac tromethamine	8,16%	0,37%	5114	551
Dicumarol	8,16%	0,40%	3586	1447
Atorvastatin	8,13%	0,53%	2531	232
Etretinate	8,12%	0,37%	5224	382
Vidarabine	8,11%	0,41%	3771	908
Tioconazole	8,08%	0,42%	4264	63
Timolol maleate salt	8,08%	0,38%	4750	392
Gefitinib	8,05%	0,46%	3010	787
Raclopride	8,05%	0,41%	4338	NA
Norgestimate	8,04%	0,37%	4911	704
Rimexolone	8,03%	0,37%	5256	30
Ketoconazole	8,03%	0,37%	4940	608
Terconazole	8,02%	0,46%	3476	5
Bacitracin	8,02%	0,39%	4906	13
Venlafaxine	8,00%	0,38%	4756	312
Zopiclone	7,99%	0,38%	4850	312
Chlorpromazine hydrochloride	7,98%	0,37%	4695	803

R(-) Apomorphine hydrochloride hemihy	7,96%	0,40%	4416	204
Astemizole	7,96%	0,48%	2916	425
Prochlorperazine dimaleate	7,95%	0,37%	4892	535
Nystatine	7,94%	0,39%	4135	1114
Ethynodiol diacetate	7,89%	0,39%	4730	210
Gatifloxacin	7,87%	0,40%	4254	310
Perphenazine	7,85%	0,39%	4464	547
Dehydroisoandosterone 3-acetate	7,84%	0,37%	5094	412
Oxethazaine	7,83%	0,40%	3652	1117
Ziprasidone Hydrochloride	7,81%	0,41%	3957	485
Medrysone	7,80%	0,38%	4184	1245
Fexofenadine HCl	7,80%	0,44%	3457	280
Papaverine hydrochloride	7,80%	0,40%	4007	610
Protriptyline hydrochloride	7,79%	0,37%	5034	127
Imatinib	7,78%	0,38%	4770	230
Metixene hydrochloride	7,77%	0,39%	4399	516
Cefazolin sodium salt	7,75%	0,39%	4018	974
Cyproterone acetate	7,75%	0,39%	4679	151
Metergoline	7,73%	0,44%	3656	153
Probucol	7,71%	0,41%	3992	425
Kanamycin A sulfate	7,66%	0,41%	3823	479
Florfenicol	7,50%	0,38%	4598	341
Tyloxapol	7,47%	0,40%	4020	410
Pentamidine isethionate	7,42%	0,44%	3202	408
Dacarbazine	7,34%	0,45%	3048	408
Megestrol acetate	7,27%	0,37%	4616	279
Clofazimine	7,20%	0,46%	2644	780
Benzethonium chloride	7,13%	0,43%	2986	896
Ethaverine hydrochloride	7,11%	0,42%	3742	76
Fluspirilen	7,11%	0,46%	2738	564
Spironolactone	6,20%	0,35%	4536	293
Aripiprazole	6,13%	0,38%	3500	657
Hycanthone	5,74%	0,42%	2888	325
